# Structural insight into the membrane targeting domain of the *Legionella*  deAMPylase SidD

**DOI:** 10.1371/journal.ppat.1008734

**Published:** 2020-08-27

**Authors:** Igor Tascón, Xiao Li, María Lucas, D’anna Nelson, Ander Vidaurrazaga, Yi-Han Lin, Adriana L. Rojas, Aitor Hierro, Matthias P. Machner

**Affiliations:** 1 CIC bioGUNE, Basque Research and Technology Alliance (BRTA), Bizkaia Technology Park, Derio, Spain; 2 Division of Molecular and Cellular Biology, *Eunice Kennedy Shriver* National Institute of Child Health and Human Development, National Institutes of Health, Bethesda, Maryland, United States of America; 3 Ikerbasque, Basque Foundation for Science, Maria Diaz de Haro, Bilbao, Spain; Gifu University, JAPAN

## Abstract

AMPylation, the post-translational modification with adenosine monophosphate (AMP), is catalyzed by effector proteins from a variety of pathogens. *Legionella pneumophila* is thus far the only known pathogen that, in addition to encoding an AMPylase (SidM/DrrA), also encodes a deAMPylase, called SidD, that reverses SidM-mediated AMPylation of the vesicle transport GTPase Rab1. DeAMPylation is catalyzed by the N-terminal phosphatase-like domain of SidD. Here, we determined the crystal structure of full length SidD including the uncharacterized C-terminal domain (CTD). A flexible loop rich in aromatic residues within the CTD was required to target SidD to model membranes *in vitro* and to the Golgi apparatus within mammalian cells. Deletion of the loop (Δloop) or substitution of its aromatic phenylalanine residues rendered SidD cytosolic, showing that the hydrophobic loop is the primary membrane-targeting determinant of SidD. Notably, deletion of the two terminal alpha helices resulted in a CTD variant incapable of discriminating between membranes of different composition. Moreover, a *L*. *pneumophila* strain producing SidDΔloop phenocopied a *L*. *pneumophila* Δ*sidD* strain during growth in mouse macrophages and displayed prolonged co-localization of AMPylated Rab1 with LCVs, thus revealing that membrane targeting of SidD via its CTD is a critical prerequisite for its ability to catalyze Rab1 deAMPylation during *L*. *pneumophila* infection.

## Author summary

The frequency of Legionnaires' disease outbreaks, a serious pneumonia caused by the intracellular pathogen *Legionella pneumophila*, has increased more than 4-fold between the years 2000 and 2015 [CDC, Summary of Notifiable Diseases, United States, 2015]. *L*. *pneumophila* secretes hundreds of bacterial effectors to manipulate host cell processes. Rab GTPases, which control intracellular vesicle trafficking in eukaryotes, are a known target of *Legionella* effectors. SidM and SidD both target host cell Rab1, but with opposing activities. While SidM catalyzes the attachment of adenosine monophosphate (AMP) to Rab1, SidD catalyzes AMP removal from Rab1, a process known as deAMPylation. Here we present the crystal structure of full length SidD, including the previously uncharacterized C-terminal domain (CTD). We discovered that the CTD assumed a unique fold that was critical for membrane localization of SidD within host cells, and that this localization was dependent on two structural elements: a stretch of hydrophobic amino acid residues that represents a general membrane targeting determinant, and a two-helix bundle that functions as the specificity determinant. Deletion of either of these elements interfered with the correct localization of SidD within mammalian cells, confirming that positioning of bacterial effectors is critically important for their biological function.

## Introduction

Altering the function of host proteins through post-translational modification is a popular strategy among microbial pathogens [1]. While some modifications like ubiquitination or phosphorylation are almost universal among eukaryotic proteins, others such as phosphocholination and AMPylation are less frequent yet equally fascinating. AMPylation (also known as adenylylation) was first described in 1967 for the *Escherichia coli* enzyme GS-ATase (glutamine synthetase adenylyl transferase; GlnE) which modifies the glutamine synthetase with adenosine monophosphate (AMP) [2, 3]. More than 40 years passed before AMPylation activity was rediscovered in translocated effectors from *Vibrio parahemolyticus* (VopS) [4], *Histophilus somnii* (IbpA) [5], and *L*. *pneumophila* (SidM/DrrA) [6]. In each of these newer cases, the AMPylated host targets were Rho or Rab family small guanine nucleotide binding proteins (GTPases) that regulate a wide variety of processes in cells.

The activation state of Rab GTPases is determined by the type of guanine nucleotide they are bound to [7]. The GDP-bound form is inactive, whereas the GTP-bound conformation is active. Activation of Rab GTPases is stimulated by GDP/GTP exchange factors (GEFs), while GTPase-activating proteins (GAPs) stimulate the intrinsic GTP hydrolysis activity of Rabs, thereby promoting conversion of GTP to GDP and, consequently, Rab inactivation [7]. In their active form, Rab GTPases are anchored to membranes via geranylgeranyl lipid groups that are covalently attached to C-terminal cysteine residues, whereas inactive GTPases are chaperoned to the cytosol by GDP dissociation inhibitor (GDI) [7, 8].

*L*. *pneumophila* is a facultative intracellular pathogen that, upon inhalation of contaminated water droplets, can enter the human lung and cause Legionnaires’ pneumonia, a potentially fatal disease that primarily impacts individuals with a weakened immune system [9]. Upon phagocytosis by monocytes such as alveolar macrophages, *L*. *pneumophila* translocates close to 300 effector proteins into the host cytosol to bypass cellular defense mechanisms and establish a safe replication compartment, the *Legionella*-containing vacuole (LCV) [10, 11]. Effector translocation requires a functional type IV secretion system (T4SS) called Dot/Icm [12, 13], and interference with this process by disrupting or mutating *dot/icm* genes renders *L*. *pneumophila* avirulent, underscoring the importance of the effectors for host cell manipulation.

During infection, *L*. *pneumophila* acquires material from various membrane trafficking pathways in order to convert the originally plasma membrane-derived vacuole into a camouflaged endoplasmic reticulum (ER)-like compartment [14]. Rab GTPases control vesicle trafficking within eukaryotic cells and are, thus, frequently manipulated by intracellular pathogens, including *L*. *pneumophila* [15, 16]. Rab1 plays a key role in the transport of early secretory vesicles from the ER to the Golgi [17]. The effector SidM (or DrrA) recruits Rab1 to the LCV surface early during infection, activates it by catalyzing GDP/GTP exchange, and AMPylates it [6, 18–22]. AMPylated Rab1 is protected from inactivation by both bacterial and host GAPs and, therefore, remains in an active GTP-bound form [6]. Accumulation of Rab1 on LCVs peaks around 2 hours post infection [18, 19]. Inactivation and removal of Rab1 is initiated upon translocation of the effector SidD which deAMPylates Rab1, thereby priming it for inactivation by the *L*. *pneumophila* GAP LepB and causing its gradual removal from the LCV 4–6 hours after bacterial uptake [23, 24]. *L*. *pneumophila* mutants lacking SidD (Δ*sidD*) fail to deAMPylate Rab1, resulting in a prolonged co-localization of AMPylated Rab1 with LCVs [23, 24]. The same observation has been made for a *L*. *pneumophila* strain lacking the Rab1 GAP LepB (Δ*lepB*), showing that both SidD and LepB cooperate in the deAMPylation and subsequent de-activation of Rab1 during *L*. *pneumophila* infection [23, 24].

SidD is a protein of 507 amino acids with no significant sequence homology to other entries in the database. Using protein crystallography, we recently succeeded in obtaining structural information about the N-terminal deAMPylase domain of SidD spanning residues 37 to 350 [25]. This domain, which is sufficient to catalyze deAMPylation *in vitro*, possesses noticeable similarity to metal-dependent protein phosphatases of the PPM family, most notably human PP2Cα and bacterial PstP [25]. The catalytic site of SidD is comprised of a binuclear metal center with strong dependence on magnesium ions, which are coordinated in part by three aspartate residues (D92, D110, D326) [25]. Substitution of any of the three aspartate residues with alanine abolished the deAMPylation activity of SidD [25]. As a consequence, a *L*. *pneumophila* strain producing SidD(D92A) phenocopied a Δ*sidD* strain and displayed prolonged co-localization with Rab1 during infection [25].

The function of the carboxy-terminal domain of SidD (CTD) has remained unclear. Truncated SidD variants lacking a complete CTD failed to localize to Golgi membranes within transiently transfected COS-1 cells, suggesting that this domain contributes to SidD localization [25]. In this study, we present the crystal structures of both the CTD and full length SidD and reveal how the CTD mediates membrane targeting of SidD, a process that we find here to be critical for the protein's ability to deAMPylate its target during infection.

## Results

### Crystal structure of SidD and its CTD

To investigate the function of the SidD CTD at a molecular level, we initiated the structural characterization of the full-length protein by X-ray crystallography. Crystals that diffracted to 3.6 Å were obtained with a slightly truncated variant comprised of residues 37–507 (SidD_37-507_). At this stage, crystal derivatization using heavy-atom soaking procedures or isomorphous replacement by SeMet yielded very poor diffraction data. Further attempts at finding a molecular replacement solution using the structure of the SidD_37-350_ catalytic domain (PDB ID code 4IIP) as a search model provided a clear solution with 6 molecules per asymmetric unit that showed partial-difference electron density for the remaining C-terminal region of SidD. However, the lack of high-resolution data precluded the iterative refinement and model-building steps required to interpret the structural information. Thus, we focused on obtaining a higher-resolution crystal structure of the CTD alone. The construct encompassing amino acids 350–507 (SidD_350-507_) crystallized readily and diffracted well. The structure was solved by single-wavelength anomalous diffraction using selenium as the anomalous scatterer. The model was refined to a resolution of 2.5 Å (R_work_ 26.4%, R_free_ 28.7%) with good stereochemistry. Residues 350–367 and residues 496–507 have been excluded from the final model as they were located in areas of poor-quality electron density and were most likely disordered. The statistics for data collection and refinement are summarized in S1 Table.

**Fig 1:**
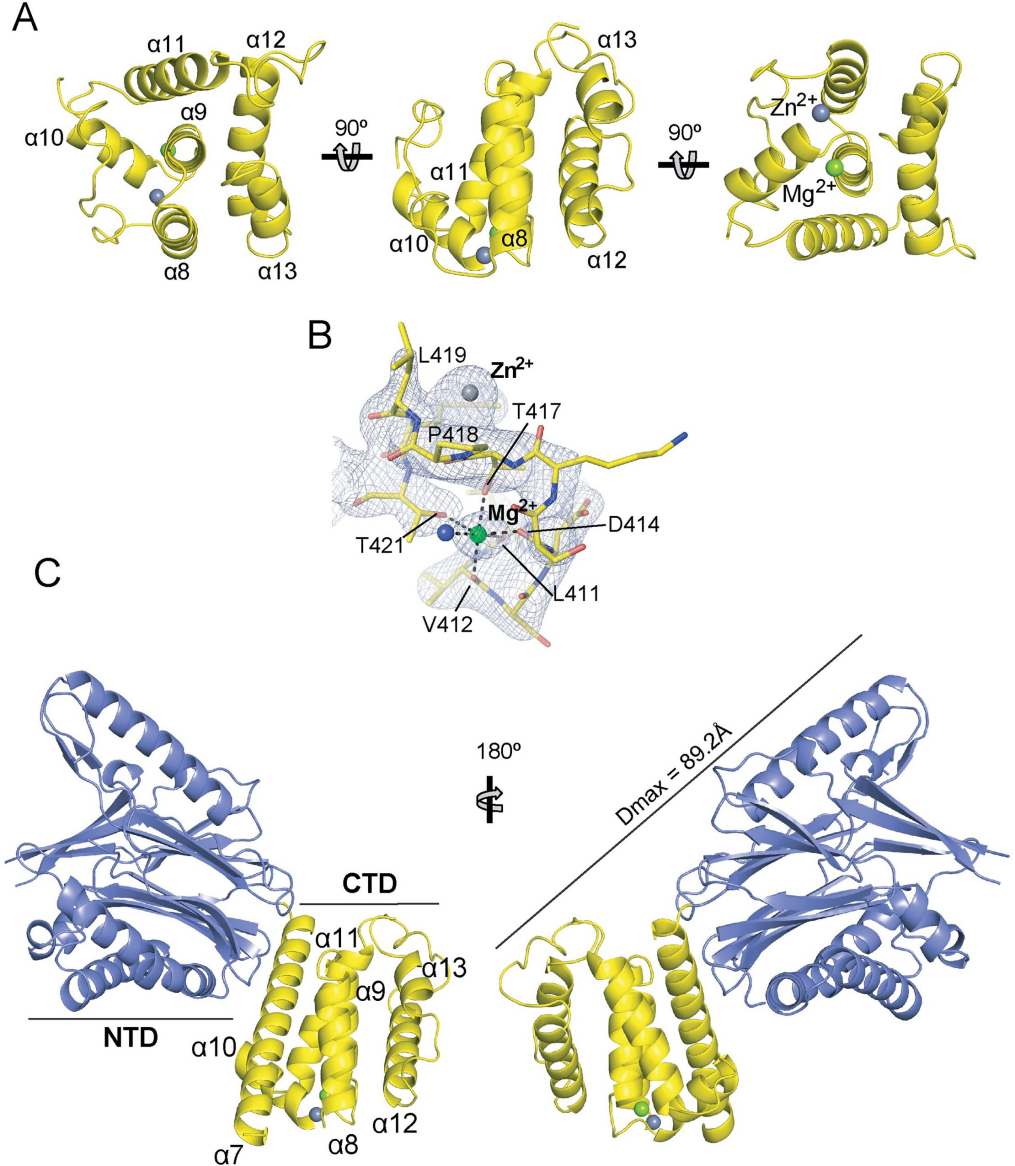
Crystal structure of SidD. (A) Crystallographic structure of the CTD of SidD, residues 350–507, shown in cartoon ribbon format in three orthogonal orientations. Nomenclature for helices continues the layout previously described for the N-terminal domain of SidD [25]. The zinc and magnesium ions are represented as grey and green spheres, respectively. (B) Final electron density map (2F_obs_-F_calc_ contoured at 1.5σ, blue mesh) within the area of the two metal ions showing the coordination sphere of the magnesium ion. (C) Crystallographic structure of SidD, residues 37–507, shown in cartoon ribbon format in two orientations. The N-terminal domain (NTD) is colored in slate and the CTD in yellow.

The structure of SidD_350-507_ revealed a compact helical domain where a central helix (α9) forms the hydrophobic core that is surrounded by five helices that are tilted with respect to the central one and form an antiparallel bundle (Fig 1A). The numbering of secondary structure elements (helices, loops) within the CTD is a continuation of the nomenclature previously introduced for the N-terminal domain of SidD [25]. Despite extensive *in silico* analyses, we were able to only find domains or regions with weak structural homology to SidD_350-507_ (for details see Supporting Material and S9 Fig to S13 Fig). Automated ion placement in PHENIX.REFINE identified a hexa-coordinated Mg^2+^ with the classical octahedral geometry formed by the O^δ^ atoms of T417, T421, the main chain carbonyl oxygens of L411, V412, and D414, and one water molecule (Fig 1B). In addition, upon closer inspection of this region we found a second metal ion, which despite not having a recognizable coordination shell, was identified as Zn^2+^ based on the clear Zn peaks in anomalous difference Fourier maps from data collected at the zinc edge (Fig 1B and S1 Fig). Given their selective retention during protein production in and purification from *E*. *coli*, we cannot exclude the possibility that they contribute to structural stability of SidD or possibly even a yet undefined catalytic activity (see Supplementary Information).

**Fig 2:**
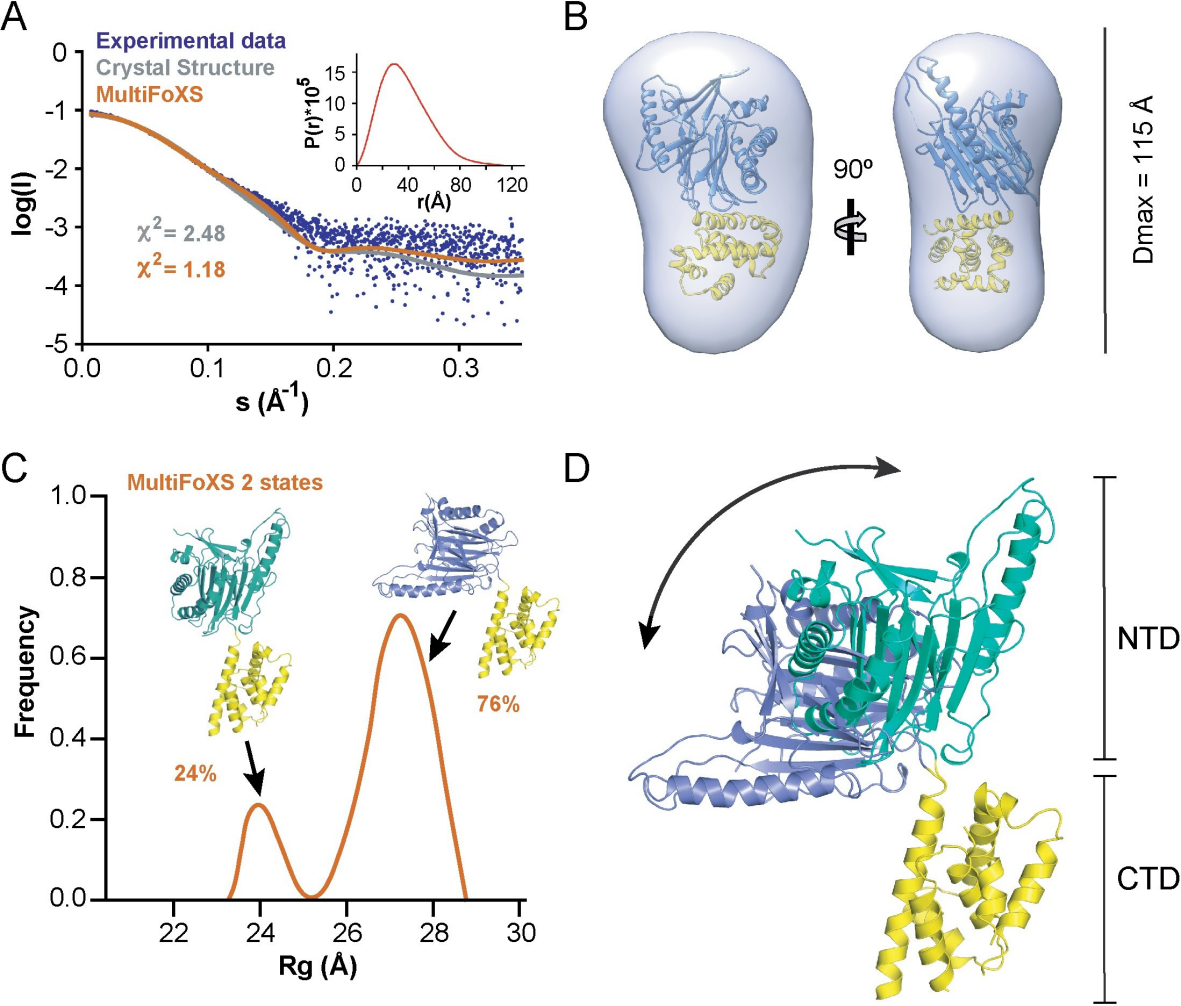
SAXS-derived conformational assemblies of SidD_37-507_. (A) Comparison of the experimental SAXS profile (blue circles) along the computed scattering from the crystallographic structure (grey line) and the profile calculated from the multi-state model obtained by the program MultiFox (orange line). The SAXS patterns are displayed as the logarithm of the scattering intensity (I) versus the momentum transfer (s). Inset: plot of the pair distance distribution function P(r). (B) Overlay of the DAMMIF-derived ab initio shape envelope with the crystal structure of SidD_37-507_. (C) MultiFoXS analyses of SidD_37-507_ SAXS data shows that two conformation ensembles are present in solution as seen from the radius of gyration (Rg) distribution. Cartoons represent the two selected conformers by MultiFoXS and their contribution percentage. (D) Superposition of the two MultiFoXS-selected conformers of SidD_37-507_ through their C-terminal domains (yellow) showing a large interdomain movement. The N-terminal domains are colored in green and slate for the conformers with 24% and 76% contribution, respectively.

With the 2.5 Å resolution structure of SidD_350-507_ on hand, we used each of the individual structures (SidD_37-350_, PDB ID code 4IIP and SidD_350-507_, current work) as separate ensembles for the molecular replacement-based solution of the aforementioned 3.6 Å SidD_37-507_ structure. Residues 85–88, 269–276, 365–382 and 495–507 were not well-defined in the electron density map and could not be modeled. A summary of the data collection and refinement statistics is given in S1 Table. The six SidD_37-507_ molecules present in the asymmetric unit displayed identical structures with an L-like shape formed by two domains in nearly perpendicular orientation (Fig 1C). We used small-angle X-ray scattering (SAXS) to evaluate the overall structure of SidD in solution. The ab initio molecular envelope calculated from the SAXS data revealed a similar L-shaped structure but with a maximum diameter D_max_ of 115 Å, which differed from the calculated value of 89.2 Å for the crystal structure. Indeed, the SidD_37-507_ crystallographic structure exhibited a low fit with the observed solution scattering curve (*χ*^2^ = 2.48) supporting the hypothesis of interdomain flexibility (Fig 2A and 2B). Given that the scattering curve might represent the average of a mixture of conformations coexisting in solution, we explored this possibility by multi-state modeling with SAXS profiles (MultiFoXS) [26]. This approach allowed the deconvolution of the scattering pattern of SidD_37-507_ into two subsets of conformations that best reproduced the SAXS profile. A superposition of these structures through the CTD showed significant tumbling of the N-terminal deAMPylase domain, suggesting that SidD is not a rigid molecule (Fig 2C and 2D).

### An exposed hydrophobic loop mediates membrane association of SidD

**Fig 3:**
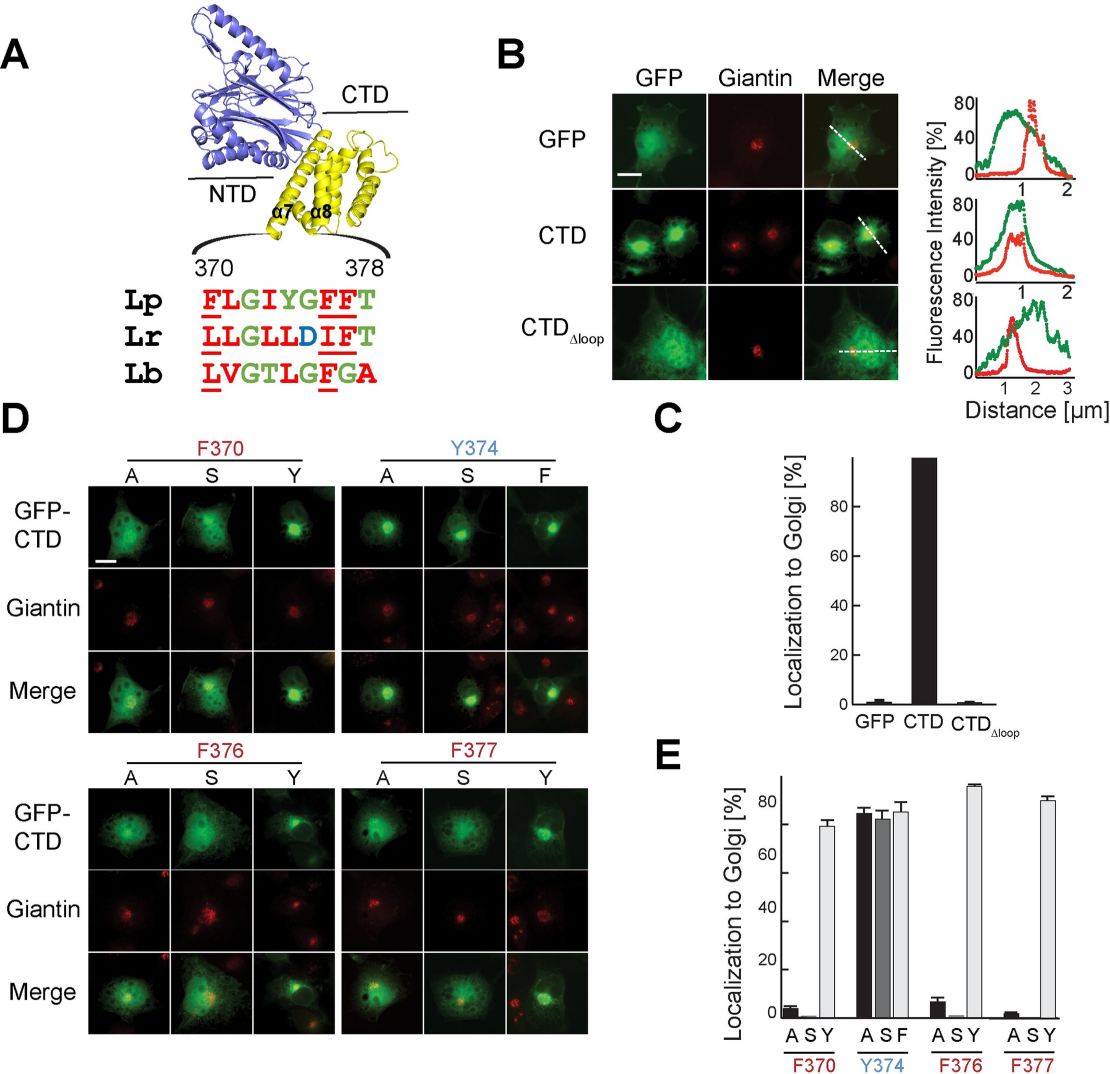
A hydrophobic loop within the CTD is required for SidD localization. (A) Schematic representation of the position and composition of the loop_CTD_ and alignment with SidD homologs from *L*. *rowbothamii* (Lr) and *L*. *belliardensis* (Lb). Hydrophobic residues within the loop region are colored in red. NTD, N-terminal domain; CTD, C-terminal domain. Numbers indicate amino acid positions. (B) Intracellular localization of CTD variants. Transiently transfected COS-1 cells producing either GFP (control) or GFP-CTD variants were fixed and stained using an antibody directed against the Golgi marker protein giantin (*middle*). Merged images show SidD proteins in green and giantin in red. Scale bar, 10 μm. Line scans (*right*) indicate pixel intensity of the green (GFP) and red (giantin) fluorescent signals along the dashed lines (distance in μm). (C) Quantification of (B) showing percentage of cells with SidD enrichment at the Golgi compartment. Numbers are results from at least 100 cells per sample and experiment. The graph represents the average of three biological replicates. (D) Effect of aromatic residue substitutions on intracellular localization of the CTD. Transiently transfected COS-1 cells producing either GFP (control, see panel B) or the indicated GFP-CTD mutants were chemically fixed, and stained for giantin to label the Golgi. The localization of CTD was evaluated by fluorescence microscopy. Amino acid substitutions at position F370, Y374, F376, and F377 were as follows: alanine (A), serine (S), tyrosine (Y), phenylalanine (F). Scale bar, 10 μm. (E) Quantification of (D) scoring cells with colocalization of GFP-CTD and giantin. The graph represents the average of at least 100 transfected cells from three biological replicates.

We previously found that the CTD is responsible for the localization of exogenously produced GFP-SidD to the perinuclear region, primarily membranes of the Golgi [25]. The molecular mechanism by which the CTD recognizes and binds to Golgi membranes, but not to other cell organelles, has remained unclear. Unlike many other *L*. *pneumophila* effectors, purified SidD showed no binding to phospholipids [25] (S2 Fig), suggesting the existence of a different mode of membrane targeting. Upon closer examination of the CTD sequence, we noticed a stretch of hydrophobic residues between helices α7 and α8 (370-FLGIYGFFT-378; loop_CTD_) that was unstructured in the crystal structure of SidD37-507, indicative of intrinsic flexibility, and that could constitute a potential membrane anchor for SidD (Fig 3A).

To confirm the importance of loop_CTD_ for SidD membrane association within mammalian cells, a loop_CTD_ deletion mutant (GFP-CTDΔ_loop_) was generated, and its localization was determined in transiently transfected COS-1 cells (Fig 3B and 3C). In comparison to GFP-tagged CTD which showed the aforementioned co-localization with the Golgi marker giantin [25], GFP-CTDΔ_loop_ failed to localize to the perinuclear Golgi region and, instead, displayed a cytosolic distribution pattern similar to that of GFP (control). To further understand the molecular details of how the loop_CTD_ mediates membrane association, we studied the importance of individual amino acids for this process. Notably, four of the nine loop_CTD_ residues had bulky aromatic side chains (underlined in 370-FLGIYGFFT-378) (Fig 3A) capable of associating with lipid bilayers by inserting into their hydrophobic core made from hydrocarbon tails. While phenylalanine tends to deeply penetrate into the hydrophobic core of membranes, tyrosine and tryptophan assumes a saddle-like distribution preferentially at the lipid-water interface [27, 28]. Sequence alignment between SidD from *L*. *pneumophila* and its only two other known homologs from *L*. *rowbothamii* (Lr) and *L*. *belliardensis* (Lb) revealed that the hydrophobic character of the loop is conserved in all three of them (Fig 3A).

To test if the aromatic residues F370, Y374, F376, and F377 within loop_CTD_ play a role for CTD membrane binding, we replaced each of them individually with either alanine (a small nonpolar residue), serine (a polar residue), or a related aromatic residue (tyrosine with phenylalanine or vice versa), and determined the localization of these SidD variants in transiently transfected COS-1 cells by fluorescence microscopy (Fig 3D and 3E). We found that substitution of F370, F376, or F377 with either alanine or serine severely disturbed Golgi localization of the mutant proteins and rendered them predominantly cytosolic. A more conservative replacement of F370, F376, or F377 with tyrosine, on the other hand, had no noticeable effect on the proteins' Golgi targeting capabilities, emphasizing the critical importance of an aromatic residue at these three positions (Fig 3D and 3E). In contrast, Y374 was dispensable for membrane binding, and its substitution with phenylalanine, alanine, or even serine had no effect on Golgi targeting of GFP-CTD (Fig 3D and 3E). Overall, these results demonstrated that the hydrophobic loop within the CTD of SidD functions as membrane-targeting determinant, and that residues with bulky hydrophobic side chains at positions 370, 376, and 377 are crucial for membrane binding.

**Fig 4:**
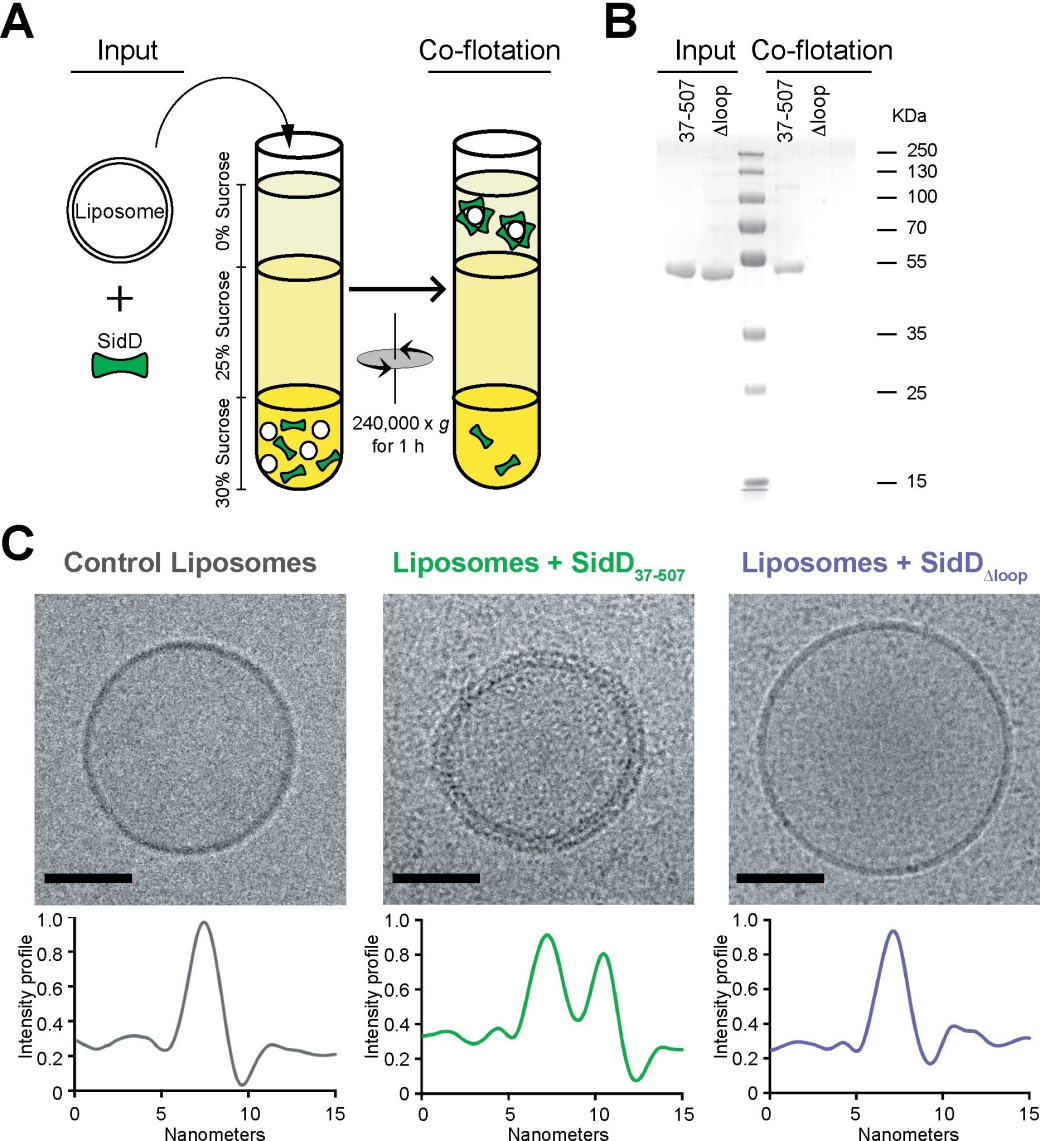
The hydrophobic loop is crucial for liposome binding of SidD during liposome flotation. (A) Illustration of the liposome flotation assay (left). (B) Coomassie blue-stained SDS-PAGE gel showing the binding of recombinant SidD_37-507_, but not SidDΔ_loop_, to free liposomes. (C) Representative cryo-EM images of a control liposome (*left*), a liposome incubated with SidD_37-507_ (*middle*), and a liposome incubated with SidDΔ_loop_ (*right*). Scale bar, 50 nm. The plot below each image represents the corresponding cross-sectional electron density profile along the perimeter of the liposome.

To further confirm the contribution of the loop_CTD_ to membrane binding, we studied the association of purified recombinant SidD_37-507_ or SidDΔ_loop_ with synthetic membranes in a co-flotation assay (Fig 4A). Upon sucrose gradient centrifugation, the majority of SidD_37-507_ was detected in the liposome-containing fraction, whereas SidDΔ_loop_ was undetectable (Fig 4B). Notably, upon cryo-electron microscopy analysis, liposomes that were incubated with SidD_37-507_ appeared fully decorated by SidD_37-507_, whereas liposomes that were incubated with SidDΔ_loop_ showed a clean bilayer as did control liposomes (Fig 4C). Finally, using surface plasmon resonance spectroscopy (SPR), we demonstrated a specific SidD-membrane interaction which yielded a dissociation constant of 4.4 μM, whilst SidDΔ_loop_, as expected, exhibited no detectable binding (S3 Fig). Importantly, circular dichroism and SAXS measurements showed that the deletion of the hydrophobic loop of SidD neither affected the structure of SidD, nor significantly altered the interdomain flexibility (S4 Fig). Together, these results demonstrate that the hydrophobic loop_CTD_ plays an integral role for association of SidD with biological membranes.

### A carboxyl-terminal pair of α-helices determines Golgi specificity of SidD

**Fig 5:**
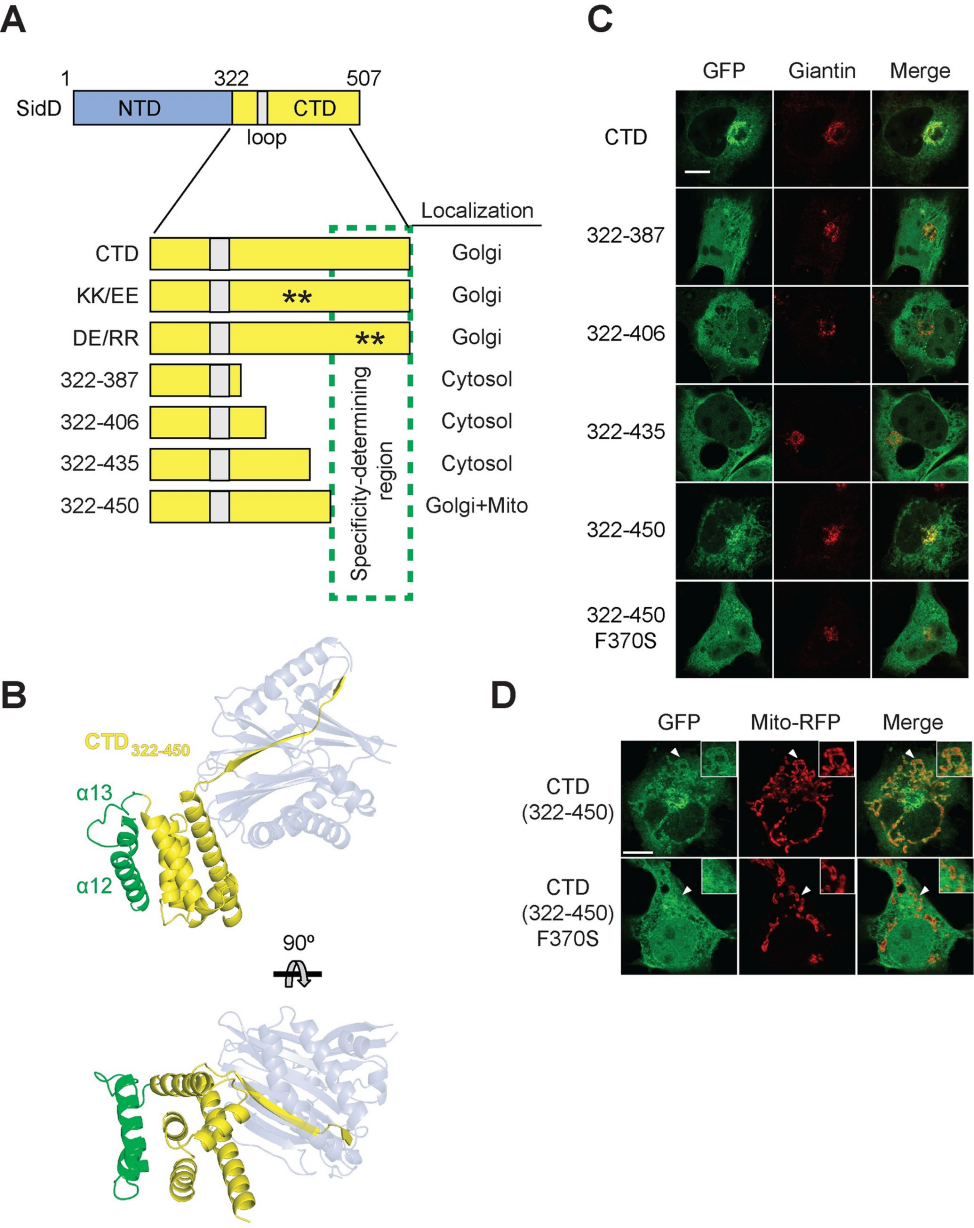
The carboxy-terminal helix bundle determines localization specificity. (A) Schematic representation of CTD and its variants. Numbers indicate amino acid positions; asterisks represent residues altered by site-directed mutagenesis. The hydrophobic loop is shown in grey, and the region required for specific localization of CTD to Golgi membranes is highlighted in green. The intracellular localization of each CTD mutant (as shown in (C)) is summarized on the right. (B) Ribbon diagram of SidD-CTD (aa 322–450) colored in orange and the C-terminal helix-turn-helix bundle is colored in green. The relative position of the deAMPylation domain is shown in transparent slate. (C) Intracellular localization of CTD variants. Transiently transfected COS-1 cells producing the indicated GFP-CTD proteins (*left*) were chemically fixed and stained for giantin (*middle*). The localization of SidD relative to giantin is shown on the right. Scale bar, 10 μm. (D) Localization of CTD(322–450) to mitochondria membranes. Transiently transfected COS-1 cells coproducing GFP-CTD(322–450) or GFP-CTD(322–450; F370S) and Mito-RFP (a mitochondria marker) were chemically fixed, and the fluorescence signal was examined by confocal microscopy. Arrowheads indicate the position of membranes magnified in the insets. Scale bar, 10 μm.

While hydrophobic interactions can significantly contribute to membrane anchoring of proteins, they provide little selectivity for a particular membrane compartment or organelle. Given the preference of exogenously produced GFP-SidD for Golgi membranes, we hypothesized that the CTD, in addition to the aforementioned membrane-targeting determinant (loop_CTD_), contained a specificity determinant that mediated preferential Golgi localization. One feature within the CTD that could constitute such a membrane specificity determinant were two patches of charged residues; a positively charged patch composed of residues K416 and K433 adjacent to the loop_CTD_, and a negatively charged patch containing D464 and E467 in helix α12 (S5A Fig). Electrostatic interactions often provide selectivity to a protein’s membrane localization and orientation by pairing with oppositely-charged lipid head groups, thus favoring interaction with specific types of membranes [29, 30]. To test if the charged patches near the loop_CTD_ were responsible for SidD’s preference for Golgi membranes, we performed charge inversion through site-directed mutagenesis, replacing either K416 and K433 with glutamates (KK/EE) or D464 and E467 with arginine residues (DE/RR). The localization of these GFP-tagged CTD mutants was examined in transiently transfected COS-1 cells. Notably, charge inversion had no noticeable effect on the extent to which the signal of either GFP-CTD(KK/EE) or GFP-CTD(DE/RR) overlapped with that of the Golgi marker giantin (Fig 5A, S5B Fig), suggesting that neither of the two charged patches made any major contribution to the enrichment of SidD on Golgi membranes or to the protein’s general membrane binding capacity.

Next, we systematically shortened SidD-CTD from its C-terminal end (Fig 5A) and determined the localization pattern of these truncated variants in transiently transfected COS-1 cells in order to identify a possible membrane specificity determinant. Despite containing the complete loop_CTD_ region, the CTD variants ending at position 387, 406, or 435, showed a cytosolic distribution pattern similar to GFP (Fig 5C), suggesting that they no longer bound to membranes, most likely because of folding or stability issues. In contrast, CTD(322–450), which lacked the carboxy-terminal two alpha-helices, α12 and α13 (Fig 5B), still associated with cellular membranes (Fig 5C, S6A Fig). Interestingly, in addition to its enrichment on Golgi membranes, CTD(322–450) was also found on other membrane compartments, including tubular organelles, that did not stain positive for giantin, suggesting that these membranes were not the Golgi. Upon co-production of GFP-tagged CTD(322–450) with a variety of organellar markers in transiently transfected COS-1 cells, we detected a substantial colocalization between CTD(322–450) and the mitochondria marker Mito-RFP (Mito-red fluorescence protein) (Fig 5D). These results suggested that the C-terminal two alpha-helices (α12 and α13) that are missing in CTD(322–450) were responsible for the selective enrichment of SidD on membranes of the Golgi, while the loop_CTD_ functioned as general membrane anchor that is insufficient to distinguish lipid bilayers of different composition. Consistent with this model, we found that substitution of F370, one of the residues critical for loop_CTD_ membrane binding (Fig 3D), with serine resulted in the failure of CTD(322–450; F370S) to associate with either Golgi or mitochondria membranes (Fig 5C and 5D and S6A Fig). Similar results were obtained in transiently transfected Hela cells, both by microscopy and subcellular fractionation, where F370S substitution rendered CTD(322–450) mostly cytosolic (S6 Fig). Taken together, our data revealed that the CTD mediates localization of SidD to membranes using a dual-binding mode that combines a general membrane-binding determinant in form of the loop_CTD_ with a specificity determinant represented by a helix-turn-helix unit at the C-terminus.

Despite extensive efforts using a variety of protein-protein interaction approaches, we were unable to identify any proteinaceous host cell factor that stably interacted with CTD. Since lipid-protein overlay assays had already excluded charged phospholipids as possible SidD ligands [25] (S2 Fig), we performed sequence alignment among the known SidD homologs and identified five conserved residues within the C-terminal two-helix bundle (L469, K472, D484, I485, L491) that are surface-exposed and available to aide in possible ligand interaction. Site-directed mutagenesis of a cluster of four of these residues, either individually or combined, had no obvious effect on Golgi localization of CTD within transiently transfected cells (S7 Fig), suggesting that membrane selectivity may involve interactions through additional CTD residues.

### SidD localizes to the surface of Legionella vacuoles

**Fig 6:**
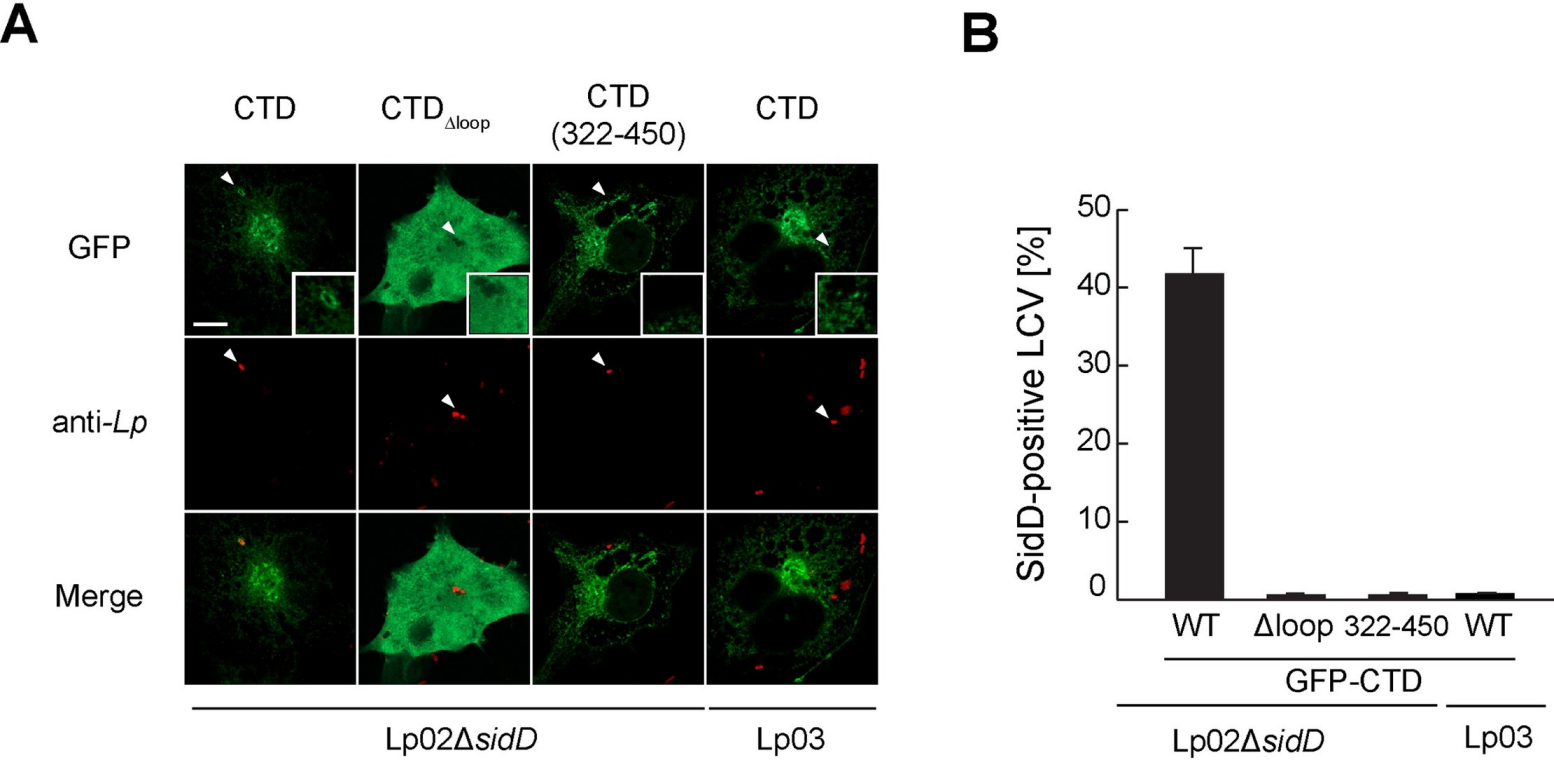
SidD localizes to LCVs via its loop_CTD_. (A) GFP-CTD localizes to the surface of LCVs. Transiently transfected COS-1 cells producing indicated GFP-CTD variants were challenged with *L*. *pneumophila* for 2 hours. Intracellular bacteria were labeled using anti-*Legionella*-specific antibody followed by TexRed-conjugated secondary antibody. GFP-CTD localization was examined by fluorescence microscopy. White arrowheads indicate the position of bacteria magnified in the insets. Scale bar, 10 μm. (B) Quantification of (A) scoring SidD-decorated LCVs. Values are an average of at least 50 LCV compartments from three experimental replicates.

A *L*. *pneumophila* Δ*sidD* mutant is defective for the timely removal of Rab1 from LCVs [23, 24], suggesting that during infection the surface of the LCVs is a site of SidD function. Nonetheless, earlier efforts to detect translocated SidD on LCVs by immunofluorescence microscopy have been unsuccessful [23, 24], most likely because the levels of endogenously produced SidD were below the detection limit. To experimentally confirm the selectivity of SidD for LCV membranes, we exogenously produced GFP-tagged CTD, CTDΔ_loop_, or CTD(322–450) in transiently transfected COS-1 cells and then challenged these cells with *L*. *pneumophila*. If LCV membranes can be recognized by the CTD, then GFP-tagged SidD should localize to the surface of these compartments. To reduce possible interference by endogenously produced SidD, a *L*. *pneumophila* Δ*sidD* strain was used for these studies, while the T4SS-deficient mutant strain (Lp03) that cannot establish a replication compartment and, instead, is delivered to lysosomes, was included as control (Fig 6). While GFP-CTD accumulated as a ring-shaped green halo about LCVs containing *L*. *pneumophila* Δ*sidD* but not LCVs containing the avirulent strain Lp03 (Fig 6), no enrichment of GFP-CTDΔ_loop_ was observed around either type of LCV (Fig 6), showing that SidD has the capacity to recognize and bind to LCV membranes containing virulent *L*. *pneumophila* in a process that requires the loop_CTD_ as membrane binding determinant. Notably, despite its ability to associate with host membranes, GFP-CTD(322–450) failed to accumulate on LCVs (Fig 6), suggesting that the C-terminal α-helix bundle determines membrane specificity of SidD during *Legionella* infection.

### Localization to *Legionella* vacuoles is critical for SidD function during infection

**Fig 7:**
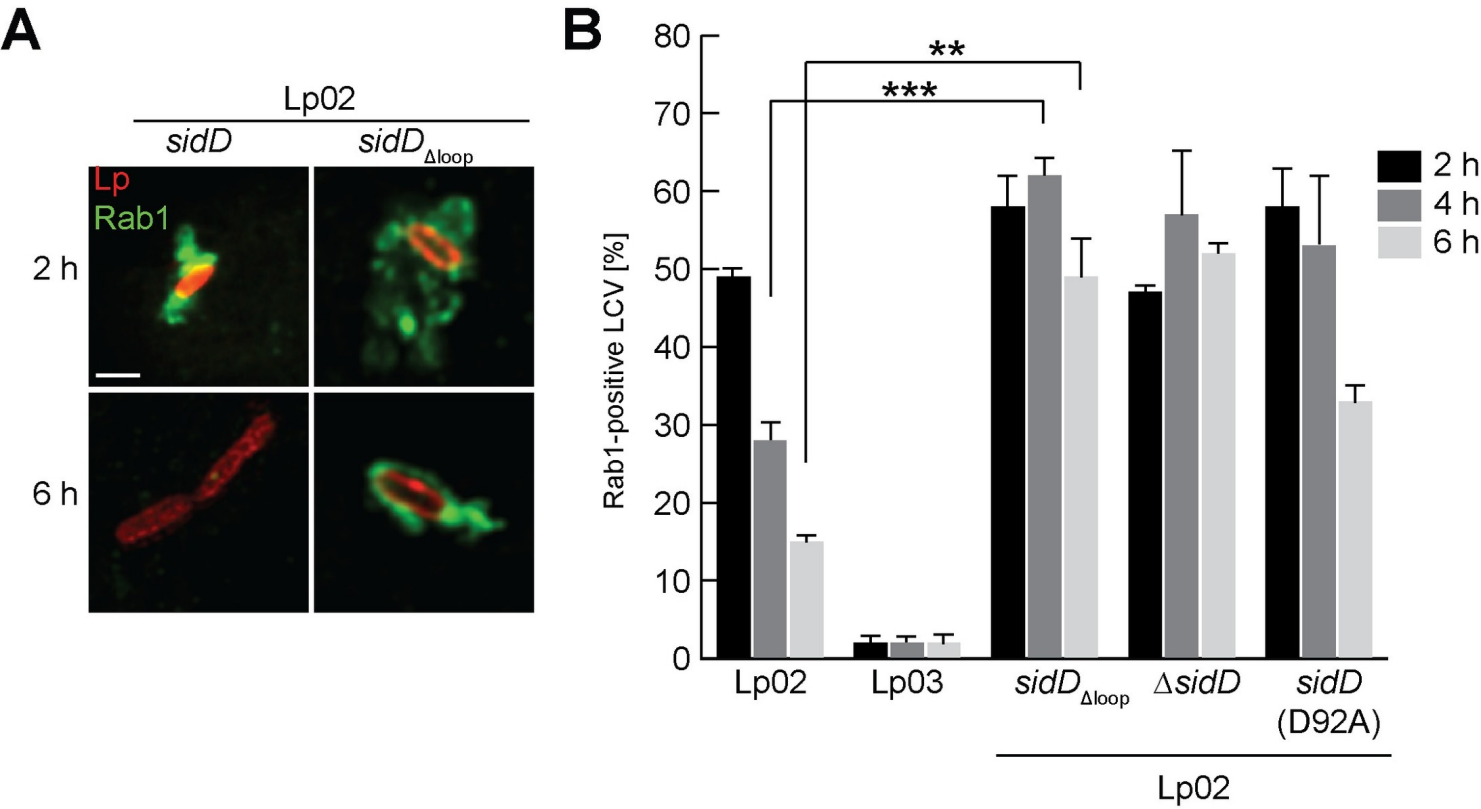
Membrane association of SidD is critical for Rab1 deAMPylation. (A) Rab1 dynamics on the LCV surface. Bone marrow macrophages challenged with the indicated *L*. *pneumophila* strains were chemically fixed at the indicated time points. Rab1 was detected by indirect immunolabeling using a Rab1B-specific antibody followed by secondary Alexa Fluor 488-conjugated antibody. *L*. *pneumophila* was stained with anti-*Legionella*-specific antibody and TexRed-conjugated secondary antibody. Cells were examined by confocal microscopy showing *L*. *pneumophila* in red and Rab1 in green. Scale bar, 1 μm. (B) Quantification of LCVs decorated with Rab1 analyzed under (A). At least 100 LCVs were counted per sample to determine the percentage of LCVs decorated with Rab1. The graph shows the average of three independent replications. ***P <0.001, **P <0.01.

With the newly acquired ability to disturb SidD membrane binding, we set out to address another important question—whether membrane localization is required for the function of SidD during infection. Earlier studies revealed that failure of *L*. *pneumophila* to deAMPylate Rab1 results in a prolonged colocalization of Rab1 with LCVs [23–25]. This kinetic defect in Rab1 removal resulted from the inability of AMPylated Rab1 to be efficiently deactivated by the Rab1 GAP LepB. Due to the lack of molecular probes capable of directly binding to and visualizing AMPylated Rab1within cells, monitoring Rab1 dynamics has been used as a surrogate assay for examining the Rab1 AMPylation status and, thus, the activity of SidD [23–25]. To investigate the importance of the loop_CTD_ for the biological function of SidD during *L*. *pneumophila* infection, the dynamics of Rab1 was analyzed in bone marrow-derived A/J mouse macrophages infected with *L*. *pneumophila* containing a chromosomal copy of either *sidD* (strain Lp02), Δ*sidD*, or *sidD*Δ_loop_. Consistent with our earlier findings [25], Rab1 colocalized with Lp02-containing LCVs early but not late during infection (49±1% positive at 2 h, 28±3% at 4 h and 15±1% at 6 h) (Fig 7A), whereas LCVs containing Lp02Δ*sidD* showed a prolonged colocalization with Rab1 compared to Lp02 (52±2% vs 15±1%) at 6 h after bacterial uptake (Fig 7B). Similar to Lp02Δ*sidD*, vacuoles containing Lp02*sidD*Δ_loop_ showed a pronounced delay in Rab1 removal from LCV membrane 4 h post infection compared to Lp02 (62% vs 28%) (Fig 7B). Rab1 removal deficiencies were even more evident 6 h post infection for Lp02*sidD*Δ_loop_, with 49% LCV staining positive for Rab1 compared to only 15% for Lp02 (Fig 7A and 7B). A similar defect in Rab1 removal was observed in bone marrow-derived macrophages challenged with Lp02*sidD*(D92A), a strain that produced a deAMPylation-inactive protein and that is known to exhibit prolonged Rab1 retention [25] (Fig 7B). Immunoblot analyses confirmed that production of SidDΔ_loop_ had no effect on the expression level of *sidM* in *L*. *pneumophila* (S8A Fig). A beta-lactamase reporter-based translocation assay also verified that the deletion of the loop did not interfere with the delivery of SidDΔ_loop_ during infection (S8B Fig). The finding that a SidDΔ_loop_ variant phenocopied the Rab1 removal deficiency of the deAMPylation defective variant SidD(D92A) indicates that membrane association via the loop_CTD_ was of critical importance for SidD to perform its biological function, namely the deAMPylation of Rab1 surrounding *Legionella* vacuoles.

## Discussion

In this study, we provided insight into the structure and function of the *L*. *pneumophila* effector SidD, the first and thus far only known translocated effector to exhibit deAMPylation activity, and discovered an important role for the CTD as a membrane targeting module.

By solving the crystal structure of SidD_37-507_, we discovered that the protein assumed an L-like shape within the crystal lattice. The N-terminal deAMPylase domain possesses a protein phosphatase fold, while the CTD is formed by a bundle of anti-parallel alpha-helices with no apparent similarity to known structures (Fig 1A). The two domains are connected via a flexible linker and are positioned almost perpendicular to each other in the crystal lattice (Fig 1C). Our SAXS analyses subsequently revealed a relative mobility between the two domains, with 76% of the particles existing as L-shaped molecules in solution while the other 24% of the particles assumed a more extended morphology (Fig 2C). If and how this interdomain flexibility between the two domains is regulated is currently unclear, but it could facilitate the recognition of AMPylated Rab1 during infection. Rab1 is connected to membranes via its ~25 amino acid long flexible C-terminal linker termed the hypervariable domain which allows the GTPase domain to be elevated 100Å or more above the lipid bilayer [31, 32]. Having a high degree of flexibility between the catalytic domain and the localization domain could increase the sampling radius of membrane bound SidD from a two-dimensional plane to a three-dimensional space (S1 Video).

A notable feature within the CTD that caught our attention was the loop_CTD_ (region 370-FLGIYGFFT-378), which is conserved among SidD homologs (Fig 3A) and critical for membrane binding of *L*. *pneumophila* SidD (Figs 3 and 4). Deletion of the loop_CTD_ strongly attenuate the ability of SidD to associate with membranes both *in vitro* (Fig 4) and within cells (Fig 3B). Moreover, substitution of any of the three phenylalanine residues at position 370, 376, or 377 with small or non-hydrophobic residue (alanine or serine) abolished the ability of GFP-CTD to robustly localize to the Golgi apparatus in transiently transfected COS-1 cells (Fig 3D and 3E), indicating that large hydrophobic side chains play a critical role for anchoring SidD to membranes. There are a wide variety of strategies used by bacterial effector proteins to associate with membranes [33], ranging from transmembrane domains (YlfA/LegC7 and YlfB/LegC2) [34], membrane curvature-sensing domains (*L*. *pneumophila* RavZ) [35] and phosphoinositide binding domains (SidM, SidC, LidA, RavD, or RavZ to name a few) [35–40] to host-mediated ubiquitination (*Salmonella* SopB) [41] and lipidation (farnesylation, geranylation, S-palmitoylation) [42–45]. The loop_CTD_ of SidD is much shorter than a typical transmembrane domain (~20 residues long) and, thus, unlikely to cross the entire lipid bilayer. Instead, our data suggest that the loop might form an amphipathic helix or segment that inserts laterally into membranes, with F370, F376, and F377 being buried within the hydrophobic core made from the lipids' hydrocarbon chains. Replacement of either one of these aliphatic phenylalanines by tyrosine did not noticeably affect Golgi localization of the CTD, showing that the amphipathic nature of tyrosines was tolerated at those positions, probably because their hydroxyl group was facing out of the lipid bilayer towards the polar head groups. Serine residues, on the other hand, were not tolerated at amino acid positions 370, 376, and 377 likely due to their hydrophilic character, and neither were alanine residues which may not penetrate the membrane deep enough in order to provide sufficient adhesion. Short stretches of hydrophobic residues have previously been found to function as membrane localization domains in several bacterial effectors, including ExoS and ExoT (both *Pseudomonas aeruginosa*), YopE (*Yersinia pseudotuberculosis*), and SopB (*Salmonella*) (reviewed in [46]). The ~22 residue long leucine-rich membrane localization region in ExoS, ExoT, and YopE is predicted to form an amphipathic helix, with charged residues clustered on one side, hydrophobic residues on the opposite face of the helix. In ExoS, substitution of the conserved leucine residues with asparagine phenocopied the membrane targeting defect of a deletion mutant lacking the entire membrane localization domain, whereas substitutions of charged residues abolished plasma membrane localization but not perinuclear localization of ExoS [47, 48]. Thus, like ExoS, SidD relies on the combined effort of a membrane binding determinant and a specificity determinant for intracellular targeting.

Not surprisingly, deletion of the membrane localization domain from ExoS or YopE limited the ability of *P*. *aeruginosa* to efficiently ADP-ribosylate host cell Ras GTPases or of *Y*. *pseudotuberculosis* to exhibit maximum virulence [47, 49]. In macrophages infected with *L*. *pneumophila*, we made a similar observation where a mutant strain producing SidDΔ_loop_ displayed the same kinetic defect in Rab1 removal from LCVs as a Δ*sidD* strain, demonstrating that proper membrane binding of SidD was critical for its function and that failure to stably associate with LCVs interfered with the ability of SidD to efficiently deAMPylate Rab1 prior to its removal (Fig 7). Our earlier studies already hinted at the importance of membrane binding for the function of SidD, where the cytotoxicity of SidM overproduction in transiently transfected COS-1 cells was counteracted by full-length SidD but not by truncated SidD lacking the CTD membrane-targeting domain [25]. We also confirmed that exogenously produced GFP-CTD, but not GFP-CTDΔ_loop_ targeted to LCVs of virulent *L*. *pneumophila* but not to those of the T4SS-defective mutant Lp03 which is delivered to lysosomes (Fig 6), indicating that SidD can distinguish different types of vacuolar membranes.

During infection, the vacuole containing virulent *L*. *pneumophila* is gradually converted into an ER-like compartment that evades endolysosomal fusion [14, 50]. Part of that transformation process is the acquisition of certain phospholipids such as PI(4)P and the removal of others (PI(3)P) [51]. Several *L*. *pneumophila* effectors have been shown to directly modify phosphoinositides, including the effector LepB (phosphatidylinositide 4-kinase), SidF (phosphoinositide phosphatase), or SidP (phosphoinositide phosphatase) [52–54]. Other effectors such as SidM and SidC possess phosphoinositide-specific binding domains for stable association with the membrane of the LCV or surrounding organelles [37, 38, 51, 55, 56]. Although SidD did not stably interact with phospholipids *in vitro* [25], the finding that it localizes to LCVs during infection or Golgi membranes in transiently transfected cells, both organelles that contain PI(4)P, suggests that this phospholipid and/or a protein common to both compartments could contribute to the selectivity of SidD for these membranes. The fact that vacuoles containing Lp03 do not maintain a stable pool of PI(4)P may explain why they failed to attract GFP-CTD (Fig 6).

By producing truncated CTD variants in transiently transfected COS-1 cells, we discovered that the selectivity of SidD for specific membranes required a C-terminal helix-loop-helix motif (residues 451–507) of the CTD, and that deletion of α12 and α13 resulted in the failure of CTD(322–450) to selectively target the Golgi (in transiently transfected cells) (Fig 5) or the LCV (during infection) (Fig 6). Instead, the protein localized to organelles other than the Golgi, including mitochondria (Fig 5D). We hypothesize that the α12 and α13 bundle forms an interaction platform for a yet-to-be determined host cell ligand that is enriched on the LCV during infection (Fig 6) or the Golgi within transiently transfected cells (Fig 5), and that deletion of this platform causes SidD to indiscriminately insert into membranes via its hydrophobic loop. While the host ligand that directs the CTD to specific membranes awaits identification, our data provide evidence for a previously unknown dual mode mechanism for membrane targeting by a *L*. *pneumophila* effector, where the combined action of a general membrane-binding determinant and a specificity determinant directs SidD to a particular type of membrane. We speculate that this dual targeting mode allows proper SidD localization within a wide variety of amoebal species that the bacterium encounters in its natural freshwater habitat, thus providing the organism with a fitness advantage. Future studies will reveal if additional effectors from *L*. *pneumophila* or related pathogens use a similar targeting strategy within host cells.

## Materials and methods

### Strains, plasmids, and reagents

All *Legionella* strains are derivatives of *Legionella pneumophila Philadelphia-1* Lp02 (*thyA*, *hsdR*, *rpsL*) [57]. Lp03 is a T4SS-defective strain variant with a mutation in *dotA* [57], while Lp02*sidD*(D92A) encodes a deAMPylation-deficient mutant form of SidD [25]. Lp02*sidD*Δ_loop_ has a chromosomal *sidD* allele with a deletion of the sequence encoding amino acids 370 to 378. *L*. *pneumophila* was cultured in liquid AYE medium or maintained on solid CYET plates as described before [58].

Plasmids and oligonucleotides used in this work are summarized in S2 Table and S3 Table, respectively. pNPTS138D-*sidD*flank was generated by subcloning a fragment ranging from 336bp upstream to 482bp downstream of *sidD* into pNPTS138D via *Gateway* cloning. pNPTS138D-*sidD*_Δloop_ was constructed using Quickchange site-directed mutagenesis (Agilent Technologies) with 5’sidD_del370-378 and 3’sidD_del370-378 as primers and pNPTS138D-*sidD*flank as template. pGEX-6P-1-SidD-CTD was constructed by subcloning the *sidD*(322–507) fragment into the *BamH*I and *Sal*I restriction sites of pGEX-6P-1. pEGFP-CTD(322–507) was previously described [25]. Plasmids encoding GFP-tagged SidD-CTD variants and C-terminal truncations were generated by QuickChange site-directed mutagenesis using the indicated primers (S3 Table) and pEGFP-SidD-CTD as template. SidD_37-507_ and SidD_350-507_ were cloned into the bacterial expression vector pHis-Parallel2 using *Nde*I-*BamH*I and *BamH*I-*Nco*I as restriction sites and adding 5 or 6 histidines tag at the C-terminus respectively. pHis-Parallel2-SidD_Δloop_ was constructed by Gibson assembly using pHis-Parallel2-SidD_37-507_ as template and the primes SidDΔloop-up, SidDΔloop-low, Amp-up and Ampi-low. The Clontech Mito-RFP plasmid was a gift from Richard Youle (National Institute of Neurological Disorders and Stroke, National Institutes of Health, Bethesda, MD). Plasmids were introduced into *L*. *pneumophila* by natural transformation [59]. *E*. *coli* was grown in Luria-Bertani (LB) broth with antibiotics (30 μg/ml Kanamycin, 100 μg/ml Ampicilin or 100 μg/ml Chloramphenicol) where necessary.

Antibodies were purchased from Santa Cruz Biotechnology (Rab1B), Abcam (giantin) and Thermo Fisher Scientific (fluorophore-conjugated secondary antibodies). Antibody against *L*. *pneumophila* was generated in rat using formalin-killed bacteria as described before [24]. Anti-SidM antibody were described previously [21].

### Cell lines and immunofluorescence microscopy

COS-1 and Hela cells were grown in Dulbecco’s modified minimum Eagles’s medium (DMEM) supplemented with 10% FBS and incubated at 37°C in 5% CO_2_. Bone marrow-derived macrophages (BMMs) were isolated from the femurs of female A/J mice and differentiated in RPMI-1640 containing 20% FBS, 1.6 mM glutamine, 30% L-cell culture medium, and penicillin (10,000 IU/ml) and streptomycin (10 mg/ml) for one week as previously described [60].

COS-1 or Hela cells grown in 24-well plates were transfected with plasmids encoding EGFP fused SidD variants using Lipofectamine® 2000 (Life Technologies, Inc.). After overnight incubation, cells were fixed with 3.8% formaldehyde, permeabilized with cold methanol, blocked with 1% BSA and stained with anti-giantin antibody at a dilution of 1:3000. Coverslips were mounted with Prolong® Gold antifade reagent (Thermo Fisher Scientific) and imaged with a Zeiss Axio Observer Z1 inverted microscope or Zeiss LSM800 confocal microscope.

For transfection-infection assays, COS-1 transfected with plasmids encoding GFP-tagged SidD variants were challenged with the indicated *L*. *pneumophila* strains at an MOI = 50, and spun at 200g for 5 min to enhance bacteria-cell contact. After 1 h, the cell monolayers were washed three times with warm DMEM to remove extracellular bacteria and incubated in DMEM for another 1 h. The cell monolayers were chemically fixed with 3.8% formaldehyde. Cells were permeabilized with cold methanol, blocked with 1% BSA, and stained for outside bacteria using rat anti-Legionella antibody and goat anti-rat Alexa Fluor355-conjugated antibody while intracellular bacteria were labeled with goat anti-rat Alexa TexRed-conjugated antibody.

BMMs were challenged with the indicated *L*. *pneumophila* strains at an MOI = 5, fixed with 3.8% formaldehyde at the indicated time points, and stained for extracellular and intracellular bacteria as described above. The presence of Rab1 on LCV membranes was determined using protein-specific antibody and goat anti-rabbit Alexa Fluor 488-conjugated antibody. Images were taken on a Zeiss LSM800 confocal microscope with Airyscan.

### Protein production and purification

Recombinant proteins for structural analyses were produced in *E*. *coli* BL21(DE3) (Stratagene) and purified similarly to previously described methods [25]. Briefly, cells were grown in LB medium to an OD_600_ of 0.8, protein production was induced with 1 mM isopropyl-β-dithiogalactopiranoside (IPTG) at 20°C overnight. Cells were harvested by centrifugation, resuspended in TBS (150 mM NaCl, 50 mM Tris-HCl, pH 7.4 supplemented with 5 mM imidazole, 10 mM β-mercaptoethanol (BME)) and lysed at 4°C by high pressure homogenization (27 Kpsi; Constant System Ltd). All subsequent purification steps were carried out at 4°C. Insoluble material was removed by ultracentrifugation, and His-tagged proteins were purified by affinity chromatography using 10 ml of Ni-NTA beads (QIAGEN) packed in a gravity column. After extensive washing with the same loading buffer, proteins were eluted by addition of 200 mM imidazole and dialyzed in 25 mM NaCl, 10 mM BME and 50 mM Tris-HCl pH 8.5 buffer. Tobacco etch virus (TEV) protease was included in a 1/20 protease/protein ratio during the overnight dialysis to cleave the His-tag. The protein solution was loaded onto an ion exchange chromatography column (HiTrap Q HP; 5 ml; GE Healthcare) followed by an isocratic gradient from 0.025 M to 1 M NaCl in 20 column volumes. Fractions containing SidD_37-507_ or SidD_350-507_ were concentrated and loaded onto a HiLoad 16/60 Superdex 200 Gel Filtration column (GE Healthcare) or a HiLoad 16/60 Superdex 75 Gel Filtration column (GE Healthcare), respectively, that had been equilibrated with TBS supplemented with 10 mM BME. Fractions containing pure protein were pooled, concentrated to 1 mg/ml and stored at -80°C. SidDΔ_loop_ was expressed and purified as described for SidD_37-507_.

Selenomethionine (SeMet)-substituted SidD_350-507_ was produced in *E*. *coli* B834 (Stratagene), a methionine auxotroph strain, using SelenoMet medium (Molecular Dimensions) following the manufacturer’s instructions. SeMet SidD_350-507_ purification was carried out under the same conditions as that of the native protein. The efficiency of SeMet incorporation was evaluated by mass differences between native (unlabeled) and SeMet-labeled protein samples using MALDI-TOF mass spectrometry. The observed mass differences confirmed 100% selenomethionine incorporation at seven expected sites.

### Crystallization, data Collection and Structure determination

Crystals of native and SeMet substituted SidD_350-507_ were grown by hanging drop vapor diffusion at room temperature by mixing a 1∶1 ratio of protein stock (8 mg∕mL) to well solution (2.0 M NaCl, 20% glycerol, 0.1 M sodium acetate, pH 4.6). Crystals of SidD_37-507_ were grown by sitting drop vapor diffusion at room temperature by mixing a 3:1 ratio of protein stock (10 mg/mL) to well solution (2.88 M sodium formate, 0.1% Anapoe 35, 0.09 M Tris-HCl, pH 8.0). Crystals of SidD_350-507_ appeared within one week whilst crystals of SidD_37-507_ appeared after three months.

Data collection with the SidD_350-507_ SeMet-substituted crystal was carried on a Pilatus 6M detector (Dectris) on the I02 beamline at Diamond Light Source (United Kingdom). Posterior data collection at higher resolution for native SidD_350-507_ (2.5 Å) and for native SidD_37-507_ (3.6 Å) was performed on a Pilatus 6M detector (Dectris) on the PXI-X06SA MD1 beamline at Swiss Light Source (Switzerland). All diffraction data sets were processed using the XDS program [61]. The Se positions in SidD_350-507_ and the phase calculations were determined by direct methods using AutoSharp [62]. An initial model was generated with phenix.autobuild within the PHENIX suite [63]. The model was manually completed in *Coot* [64] and then refined with PHENIX with alternating rounds of manual intervention and optimization. Data collection and refinement statistics are shown in S1 Table.

### SAXS analysis

SAXS data were collected on the BL21 beamline at the Diamond Light source (United Kingdom), using the HPLC-integrated SAXS setup with a Pilatus 2M detector over an angular range *q*min = 0.004 Å^-1^ to *q*max = 0.37 Å^-1^. For SidD_37-507_ or SidD_Δloop_, a volume of 45 μl at 11 mg/ml was injected into a pre-equilibrated (25 mM HEPES pH 7.5, 300 mM NaCl and 0.5 mM TCEP) Shodex KW-402.5 gel filtration column. Scattering was recorded over the course of protein elution with a flow rate of 0.16 ml/min at 20°C. Data averaging and reduction was carried out with ScÅtter (Version 3.1r by Robert P. Rambo, Diamond Light Source, UK). Further analyses (radius of gyration; Rg, maximum distance; Dmax and particle distance distribution function; *p(r)* calculations) were carried out with the ATSAS suite package [65]. Low-resolution shape envelopes for SidD_37-507_ were determined using the ab initio bead modelling program DAMMIF [66]. Modelling was performed without the use of symmetry restraints. Twenty simulations were performed, which generated similar shapes from witch an averaged filtered structure was obtained using DAMAVER [67]. Then, this structure was refined as a fixed input core in DAMMIN to generate a final model with 795 dummy-atoms. The resulting bead model was converted to a map envelope and visualized using CHIMERA [68]. The program SUPCOMB [69] was used to compare the ab initio model with the crystallographic data whilst the program CRYSOL [70] was used to compare the 1D scattering curve with the theoretical scatter of SidD_37-507_ model. The Multifox webserver [26] was used to calculate a multi-state model of SidD_37-507_ or SidD_Δloop_ in solution. The crystallographic structure was used as input model where residues I335 to L339 where considered flexible, and then 10.000 conformations were generated by the RRT (Rapid Random Trees) algorithm sampling. The top solution corresponded to a two-state model, and there was no significant improvement in the *χ*^*2*^ scores for models of three or more states.

### Lipid overlay assay

Lipid overlay assays were performed following the manufacturer’s instructions. Briefly, membrane strips (Echelon Biosciences, cat# P-6001, P-6002) with spotted lipids were blocked with TBST + 5% BSA at room temperature for 1 h, incubated with purified SidD_37-507_ or SidM at room temperature for 1 h, and washed with TBST three times to remove unbound protein. The membrane strips were then incubated with anti-SidD or anti-SidM antibody, washed again with TBST three times, followed by incubation with HRP-conjugated goat anti-rabbit secondary antibody. The binding of protein to lipids on the membrane strips was detected by chemiluminescence.

### Circular dichroism measurements

Circular dichroism (CD) measurements were carried out with a Jasco J-810 spectropolarimeter (JASCO, Tokyo, Japan) equipped with a Peltier temperature control. CD spectra were acquired at 25°C with 0.2 nm data pitch, 50 nm/min scanning speed, 4 sec response, 4 nm band width and 5 accumulations, using a 0.1 cm path length quartz cuvette. The samples were measured at a concentration of 30 μM in 5 mM HEPES buffer pH 7.5, 75 mM NaCl and 0.5mM DTT.

### Isothermal Titration Calorimetry (ITC)

SidD_37-507_ and Rab1-CT peptide were dialyzed overnight in 150mM NaCl, 0.5mM TCEP and 25 mM HEPES pH 7.5 at 4°C. The compounds; N-Acetyl-S-geranyl-L-cysteine (AGC) and N-Acetyl-S-farnesyl-L-cysteine (AFC) were dissolved in DMSO at 25 mg/ml and subsequently diluted in the dialysis buffer. Rab1-CT peptide at 1mM was titrated into SidD_37-507_ at 10 μM in aliquots of 10 μl. AGC and AFC were titrated into SidD_37-507_ (10 μM) at 417 μM and 408 μM respectively. All ITC measurements were carried out at 25°C on a VP-ITC Microcalorimeter (MicroCal/GE Healthcare). The ITC data were processed using Origin software (OriginLab Corp., USA).

### Liposome preparation

Liposome preparation for SPR: 1,2-Dioleoyl-sn-glycero-3-phosphocholine (DOPC) dissolved in chloroform:methanol (2:1) was dried under argon to obtain a lipid film, which was hydrated with 10 mM HEPES pH 7.5, 150 mM NaCl and 1 mM DTT at a final lipid concentration of 1 mM. Small unilamellar vesicles (SUVs) were prepared by vigorous vortexing of the lipid suspension followed by sonication for 10 minutes with cycles of 5 second pulses in 5 second intervals.

Liposome preparation for flotation assay: 1,2-dioleoyl-sn-glycero-3- phosphoethanolamine (DOPE), 1,2-dioleoyl-sn-glycero-3-phospho-l-serine (DOPS), and 1,2-dioleoyl-sn-glycero-3-phosphocholine (DOPC) in a 5:3:2 molar ratio containing 0.1% of Rhod-PE were dissolved in chloroform:methanol (2:1) and dried under argon to obtain a lipid film. The film was re-hydrated with flotation buffer (FB), 150 mM NaCl, 0.5 mM TCEP and 10 mM HEPES pH 7.5, with 10% sucrose, and subjected to vortex mixing. Then 10 freeze-thaw cycles followed by mechanical extrusion through a 0.2 μm cut-off filter were carried out until the mixture become clear.

Liposome preparation for cryo-EM followed the same protocol as for the flotation assay but without the inclusion of Rhod-PE and sucrose.

### Liposome flotation assay

In a standard reaction, 100 μl liposomes (1 mM) were mixed with 25 μM SidD_37-507_ or SidD_Δloop_ in buffer FB and incubated for 15 minutes. The sample was gently mixed with a stock solution of 80% sucrose in FB to achieve a final sucrose concentration of 30%, which was placed at the bottom of an ultra-Clear^TM^ tubes (Beckman Coulter, cat. 344090) and overlaid with 600 μl buffer FB25 (25% sucrose in FB), and 100 μl buffer FB. Tubes were centrifuged at 240,000 g for 1 hour at 4°C, and the top 30–50 μl fraction was collected and used for SDS-PAGE analysis.

### Cryo-electron microscopy analysis

Samples containing liposomes incubated with SidD_37-507_, SidD_Δloop_ or no protein, were loaded on freshly glow-discharged *Quantifoil R2*/*2* grids. Vitrification was performed on Vitrobot Mark II (FEI Company, USA) maintained at 8°C and at a relative humidity close to saturation (90% rH). Five microliters of sample solutions were absorbed onto the grid for 30 seconds and blotted with filter paper. Grids were immediately plunged into a liquid ethane bath and stored under L2 until visualization. Imaging of cryoTEM samples was made on a JEM-2200FS/CR transmission electron microscope operated at 200 kV. An in-column omega energy filter helped to record images with improved signal to noise ratio by zero-loss filtering. The energy selecting slit width was set at 9 eV. Digital images were recorded on UltraScan4000 CCD camera (Gatan Inc.) under low-dose conditions at a nominal magnification of 50,000x obtaining a final pixel size of 2.7 Å/pixel. Cross-sectional membrane intensity profiles were calculated with the image processing software ImageJ [71].

### Surface plasmon resonance (SPR)

SPR data were collected using a BIAcore 3000 system (GE Healthcare) and a L1 sensor chip (GE Healthcare), which contains alkyl chains for capturing liposomes. All SPR experiments were performed in running buffer (10 mM HEPES pH 7.5, 150 mM NaCl, 1 mM DTT) at 25°C. The L1 chip surface was conditioned with two injections of a mixture of isopropanol and 50 mM NaOH at a ratio 2:3 (5 μl at 10 μl/min). After overnight dialysis at 4°C against the running buffer, a 2-fold serial dilution of SidD_37-507_ and SidD_Δloop_ was prepared (32 to 0.125 μM) in running buffer. For each concentration, 20 μM liposomes were immobilized over the chip (10 μl at 5 μl/min) and any excess unbound liposomes were removed by injecting 5 μl of 50 mM NaOH (10 μl/min flow rate). Uncovered sensor chip surface was blocked with a 0.2 mg/ml BSA (10 μl at 5 μl/min) to avoid non-specific binding. Binding experiments were carried out by injecting SidD_37-507_ or SidD_Δloop_ (40 μl at 20 μl/min) over the liposome-coated chip. The L1 chip was regenerated and stripped of liposomes after each run by injecting a mixture of isopropanol and 50 mM NaOH at a ratio 2:3 (5 μl at 10 μl/min) and 20 mM CHAPS (5 μl at 10 μl/min). No loss of sensor chip binding capacity due to regeneration occurred, and the capture of liposomes was similar in each run. The sensograms were processed using the BIAcore 3000 BiaEvaluation software (GE Healthcare). Equilibrium dissociation constants were obtained by fitting the maximum RUs reached at each SidD concentration to the steady-state affinity model, assuming a 1:1 binding stoichiometry. Each experiment was done in triplicate.

### Ethics statement

All experiments were carried out in accordance with the recommendations in the Guide for the Care and Use of Laboratory Animals of the National Institutes of Health. The animal use protocol was reviewed and approved by the Institutional Animal Care and Use Committee (IACUC) of NIH (protocol number ASP#18–084).

## Supporting information

S1 Fig Zn^2+^ identification. (TIF)

S2 Fig Lipid-protein overlay assay with SidD_37-507_. (TIF)

S3 Fig SPR sensograms of SidD_37-507_ and SidD_Δloop_. (TIF)

S4 Fig Deletion of the hydrophobic loop does not significantly alter the structure of SidD or interdomain flexibility. (TIF)

S5 Fig Two charged patches near the loop region do not affect the CTD selectivity for membranes. (TIF)

S6 Fig Subcellular localization of CTD mutants. (TIF)

S7 Fig Mutational analysis of the C-terminal α-helix bundle. (TIF)

S8 Fig Study of *Legionella* with a *sidD*Δloop allele. (TIF)

S9 Fig The catalytic triad within CTD does not affect Rab1 dynamics on LCVs. (TIF)

S10 Fig Cysteine analog and synthetic peptide binding to SidD. (TIF)

S11 Fig SidD or CTD does not affect the lipidation state of Rab1. (TIF)

S12 Fig SidD or CTD does not display lipid phosphatase activity. (TIF)

S13 Fig SidD or SidD CTD does not display phospholipase activity. (TIF)

S1 Table Data collection and refinement statistics. (DOCX)

S2 Table Plasmids used in this study. (DOCX)

S3 Table Oligonucleotides used in this study. (DOCX)

S1 Video Model of SidD-membrane association and interdomain flexibility for targeting AMPylated Rab1. (MOV)
