# Supplementary figures and images for: Structural insight into the membrane targeting domain of the Legionella deAMPylase SidD
Source: PLoS Pathog. 2020 Aug 27;16(8):e1008734. doi: 10.1371/journal.ppat.1008734 (PMC7480848; doi:10.1371/journal.ppat.1008734)

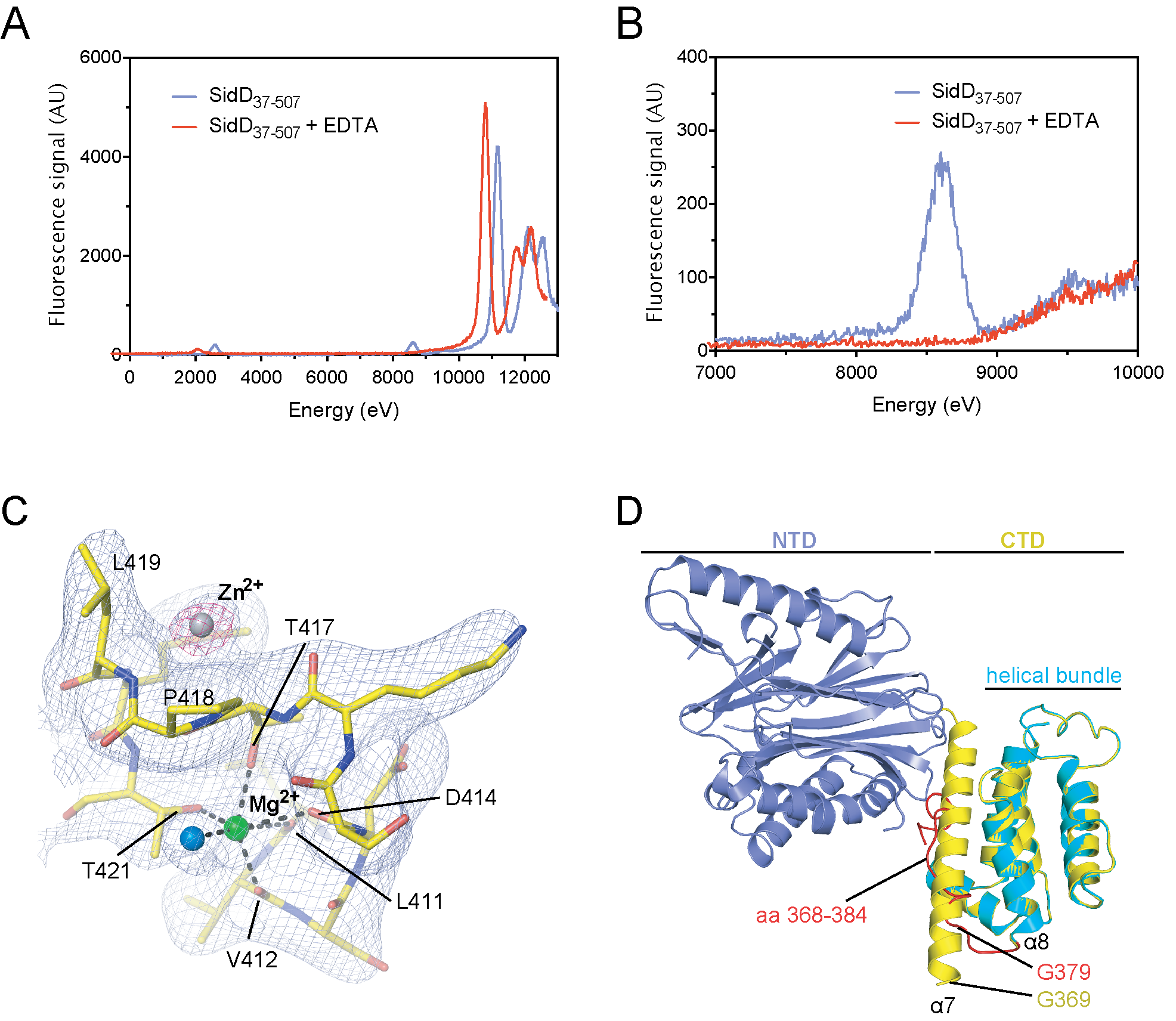

Supplement: S1 Fig — (TIF) [file ppat.1008734.s001.tif]

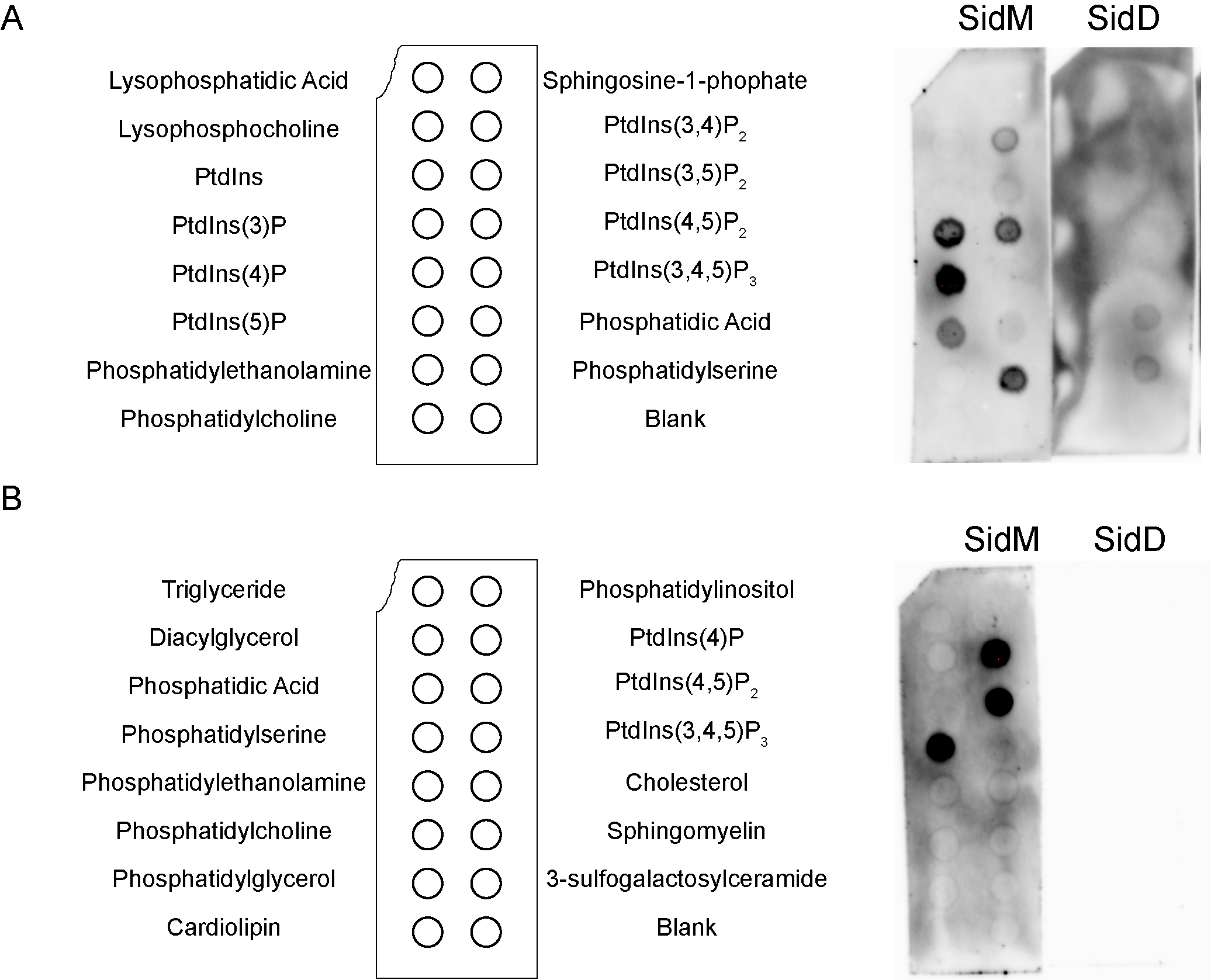

Supplement: S2 Fig — (TIF) [file ppat.1008734.s002.tif]

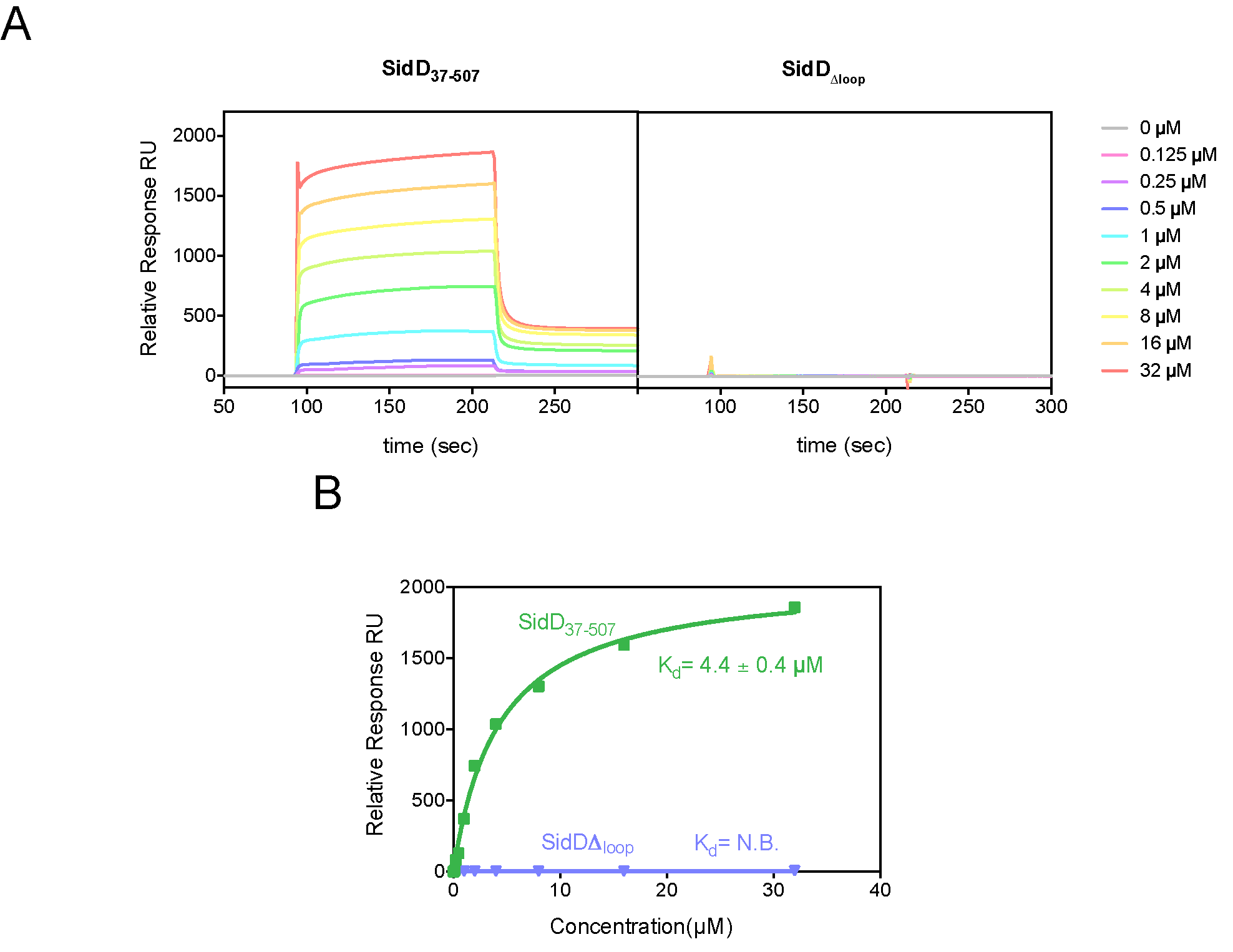

Supplement: S3 Fig — (TIF) [file ppat.1008734.s003.tif]

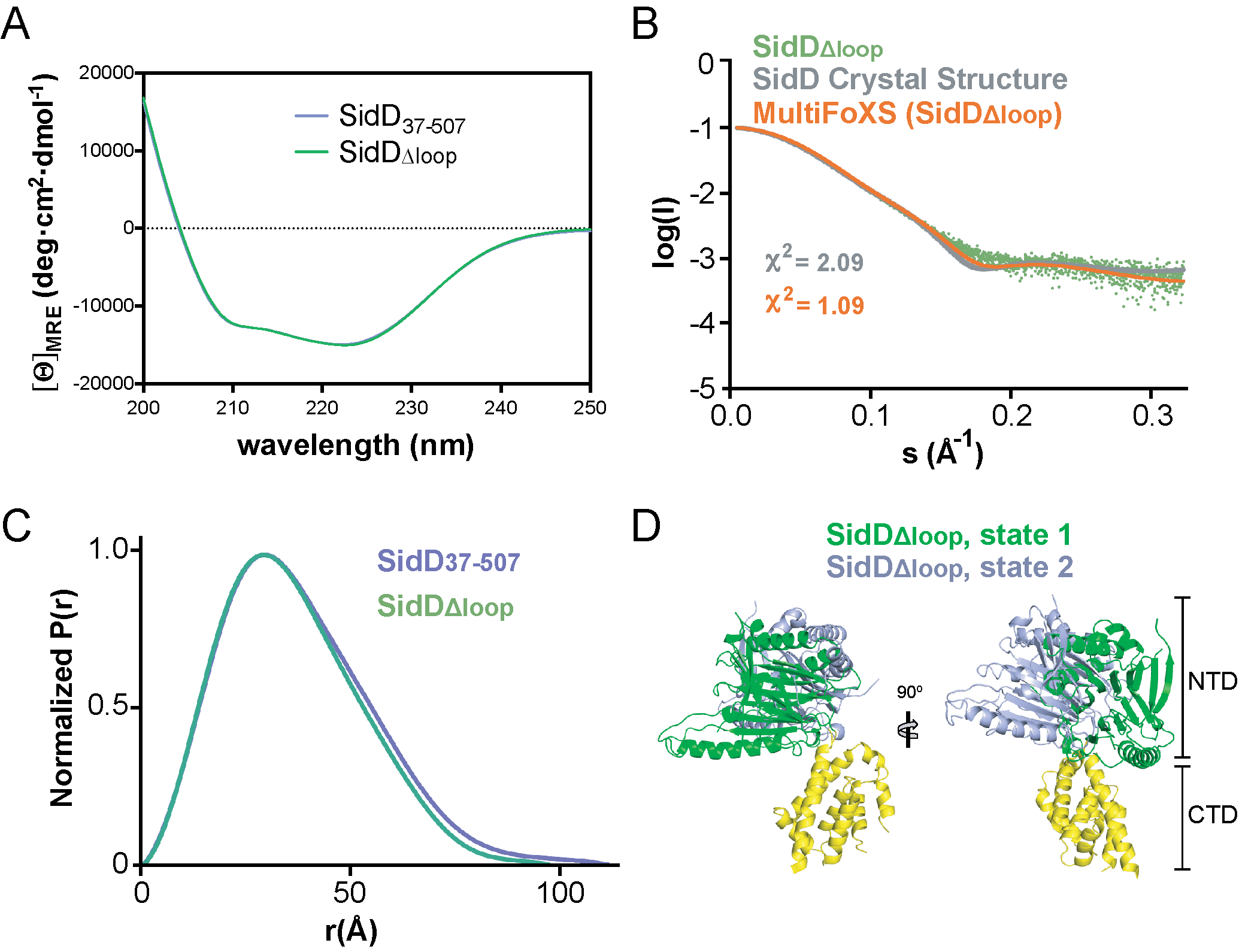

Supplement: S4 Fig — (TIF) [file ppat.1008734.s004.tif]

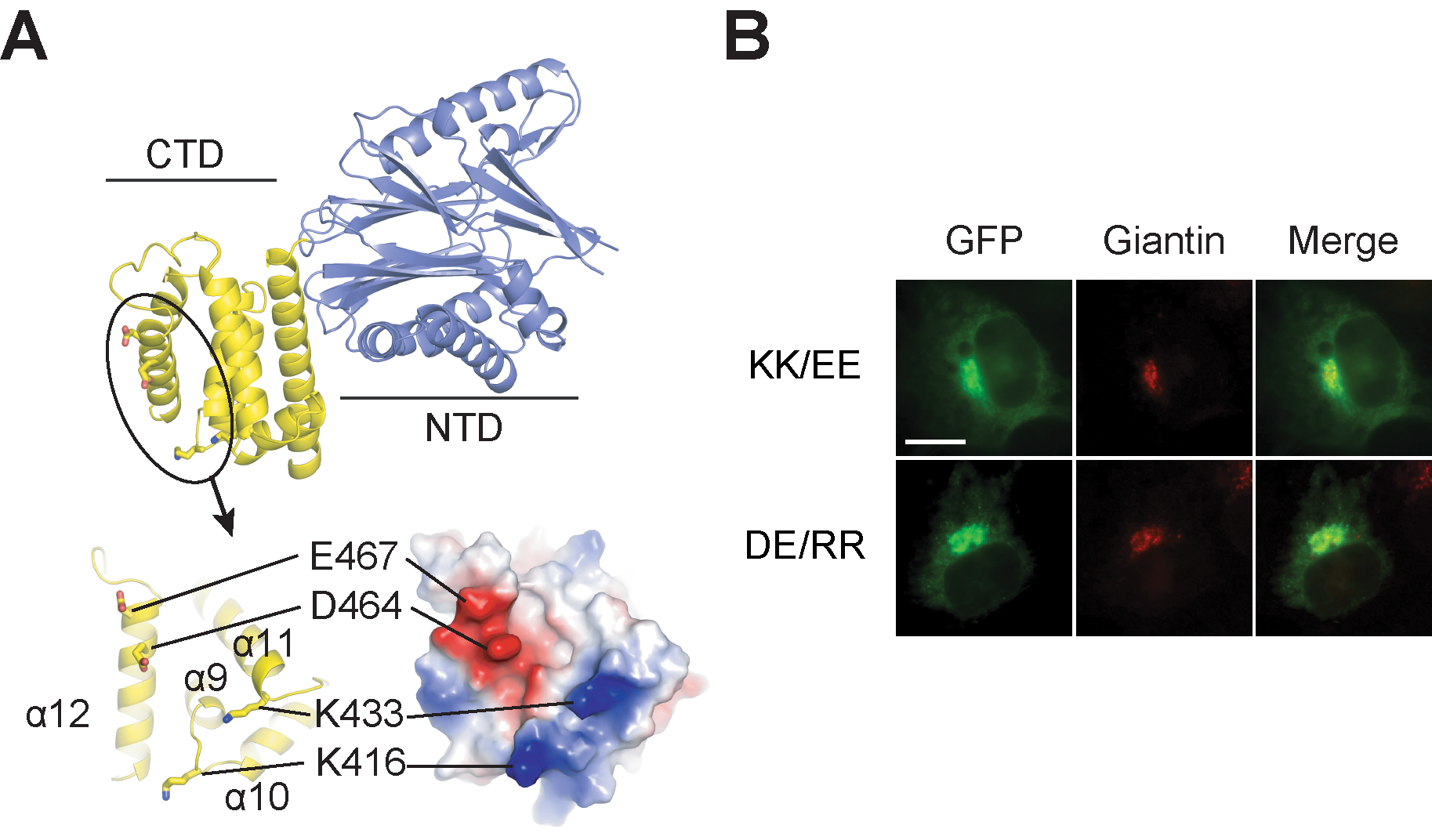

Supplement: S5 Fig — (TIF) [file ppat.1008734.s005.tif]

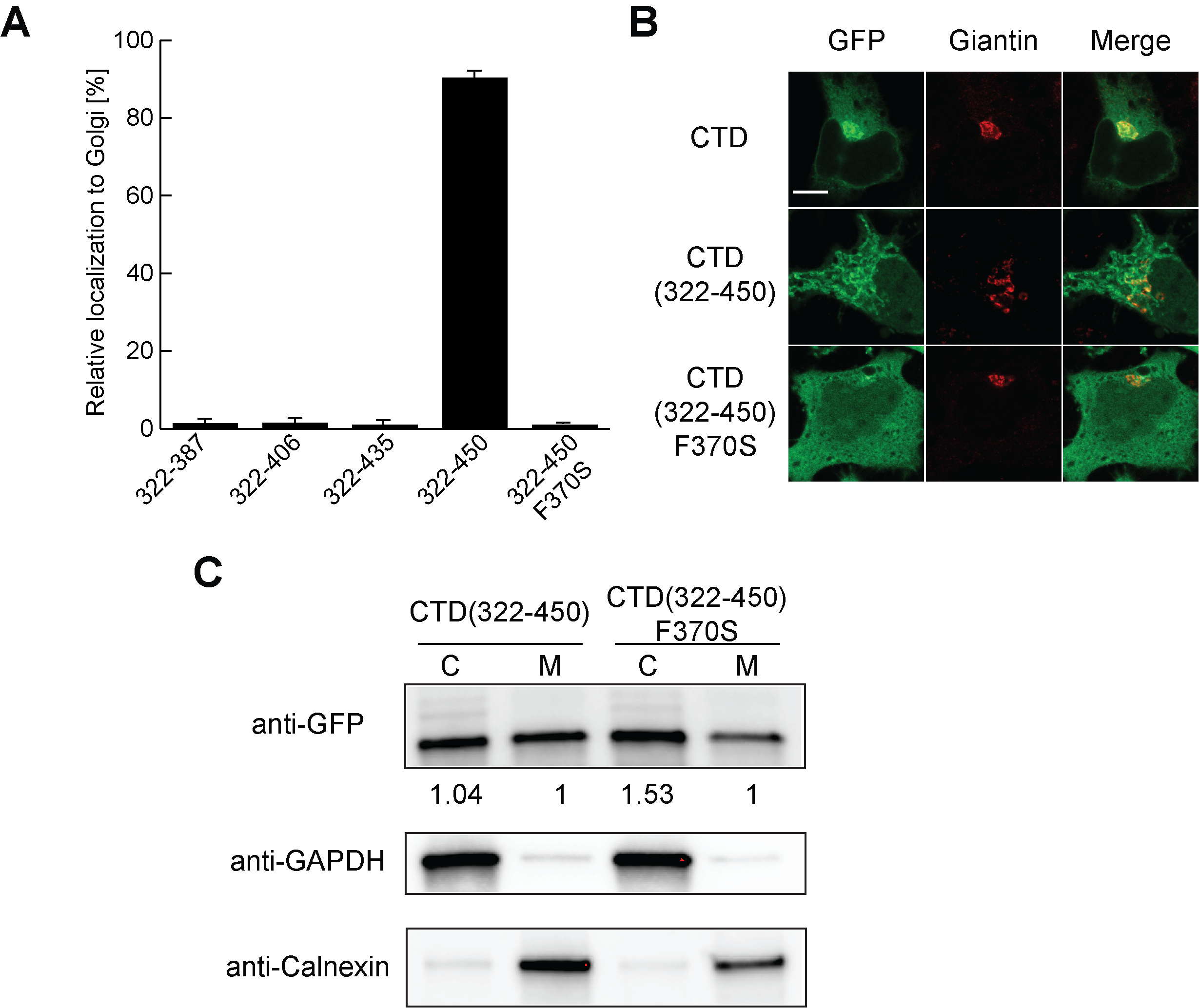

Supplement: S6 Fig — (TIF) [file ppat.1008734.s006.tif]

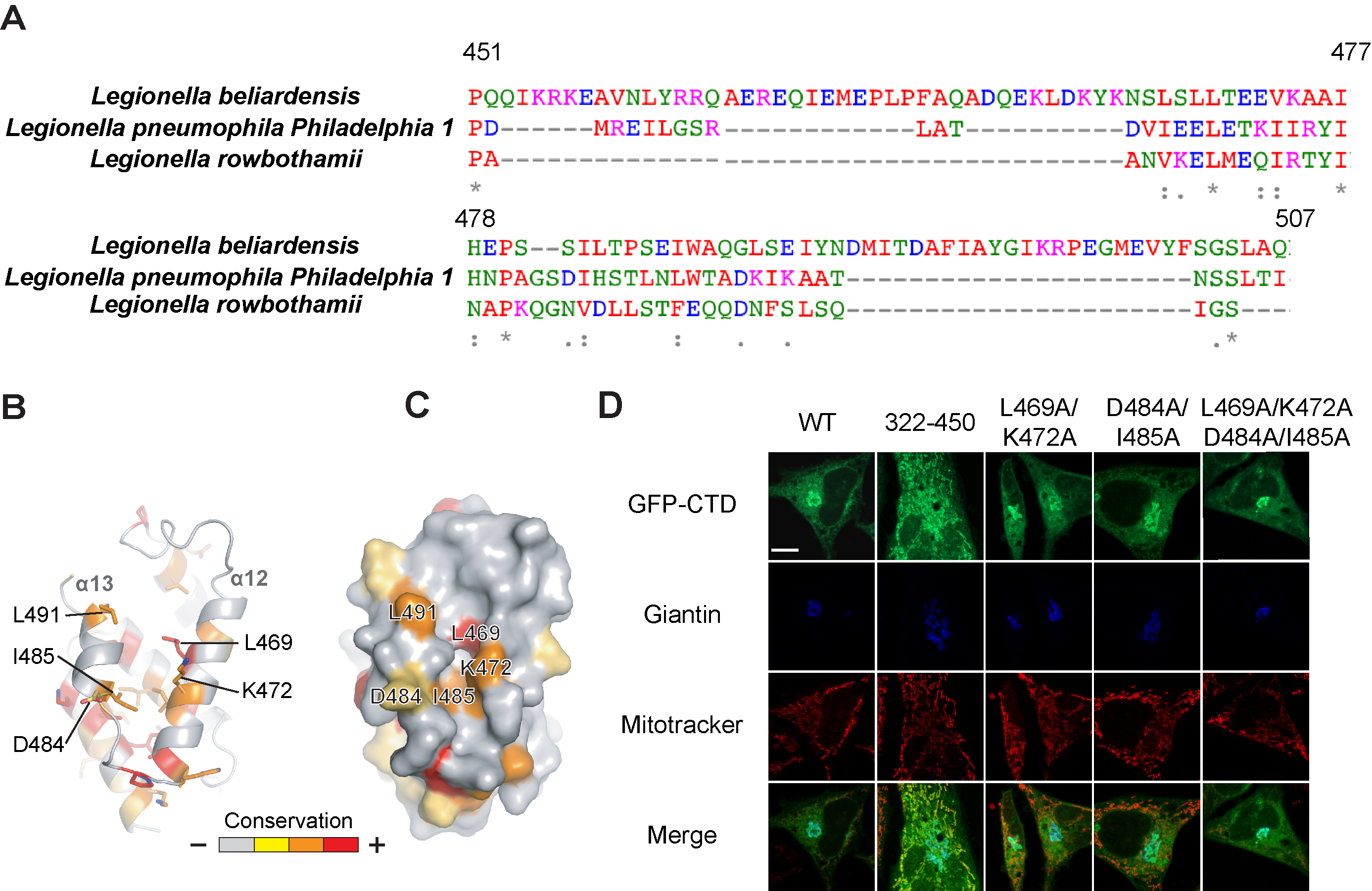

Supplement: S7 Fig — (TIF) [file ppat.1008734.s007.tif]

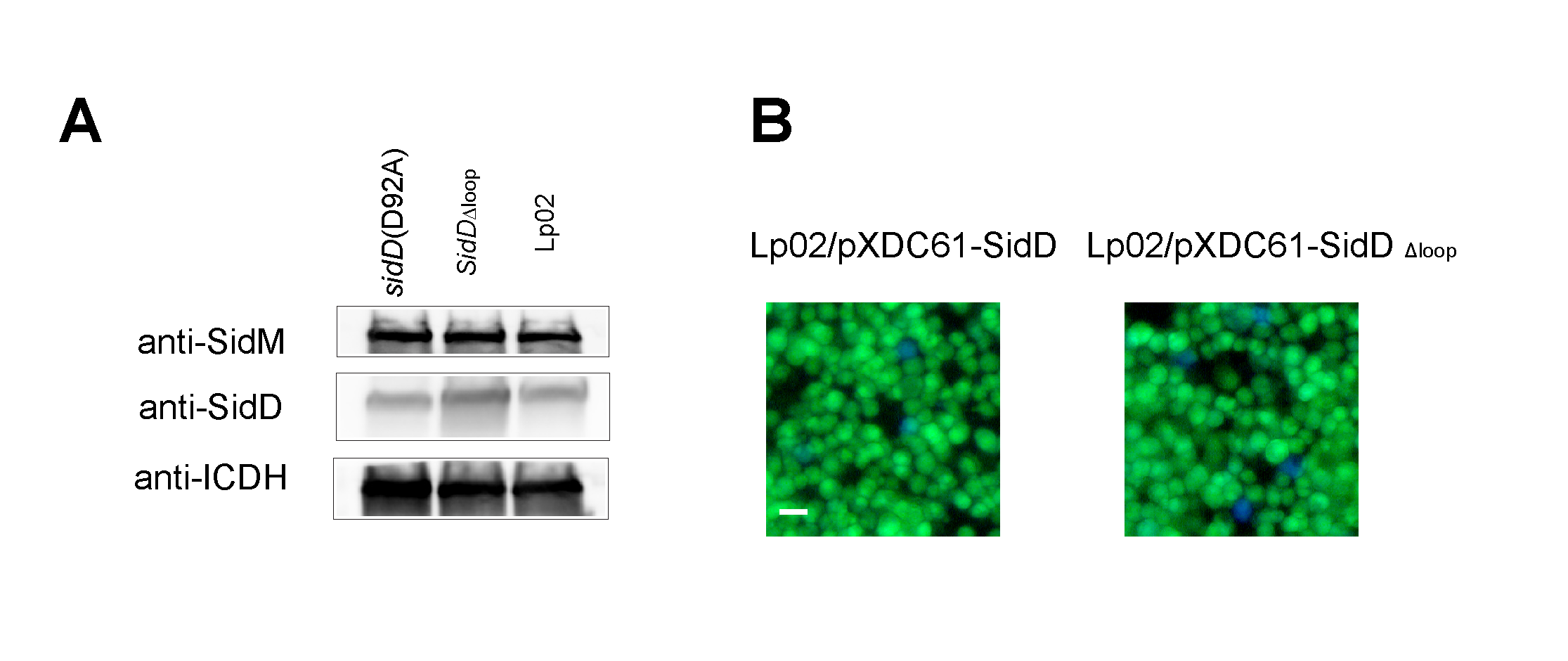

Supplement: S8 Fig — (TIF) [file ppat.1008734.s008.tif]

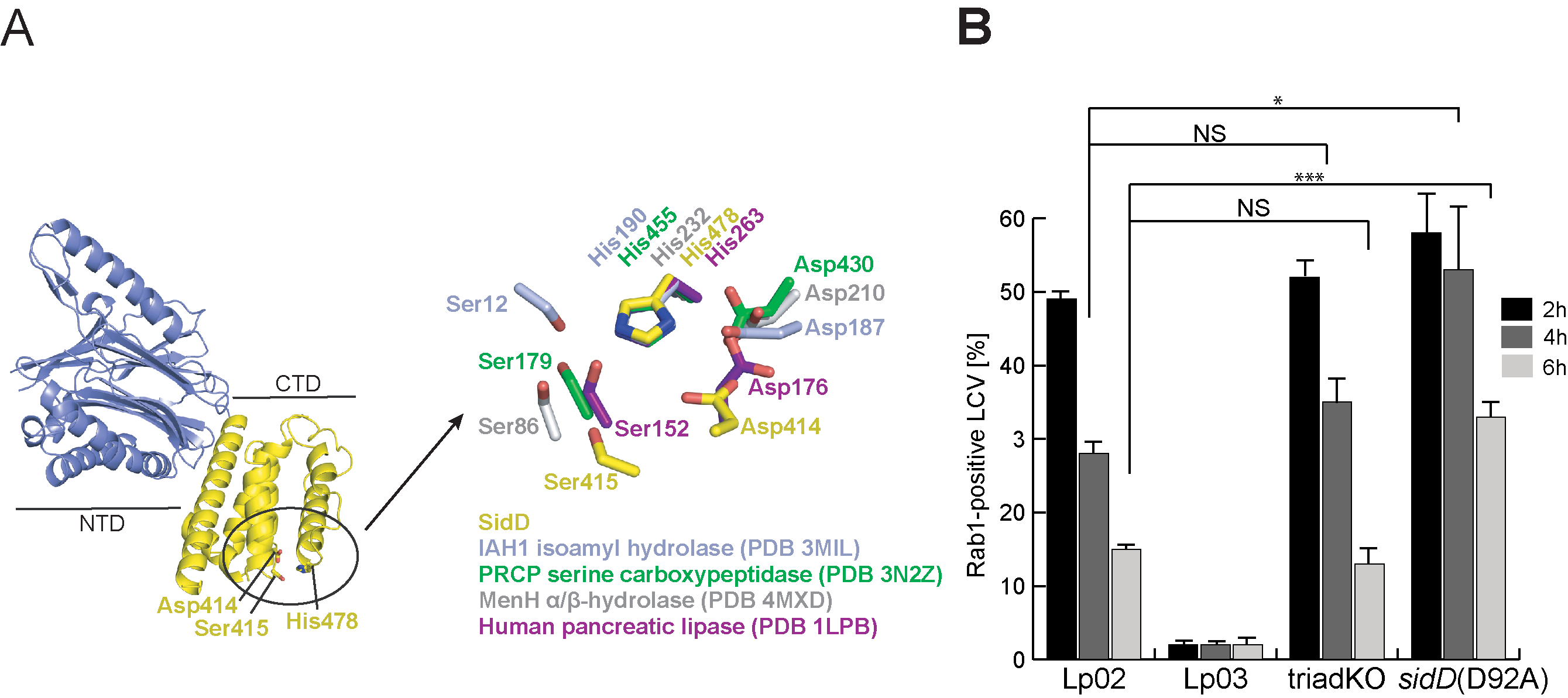

Supplement: S9 Fig — (TIF) [file ppat.1008734.s009.tif]

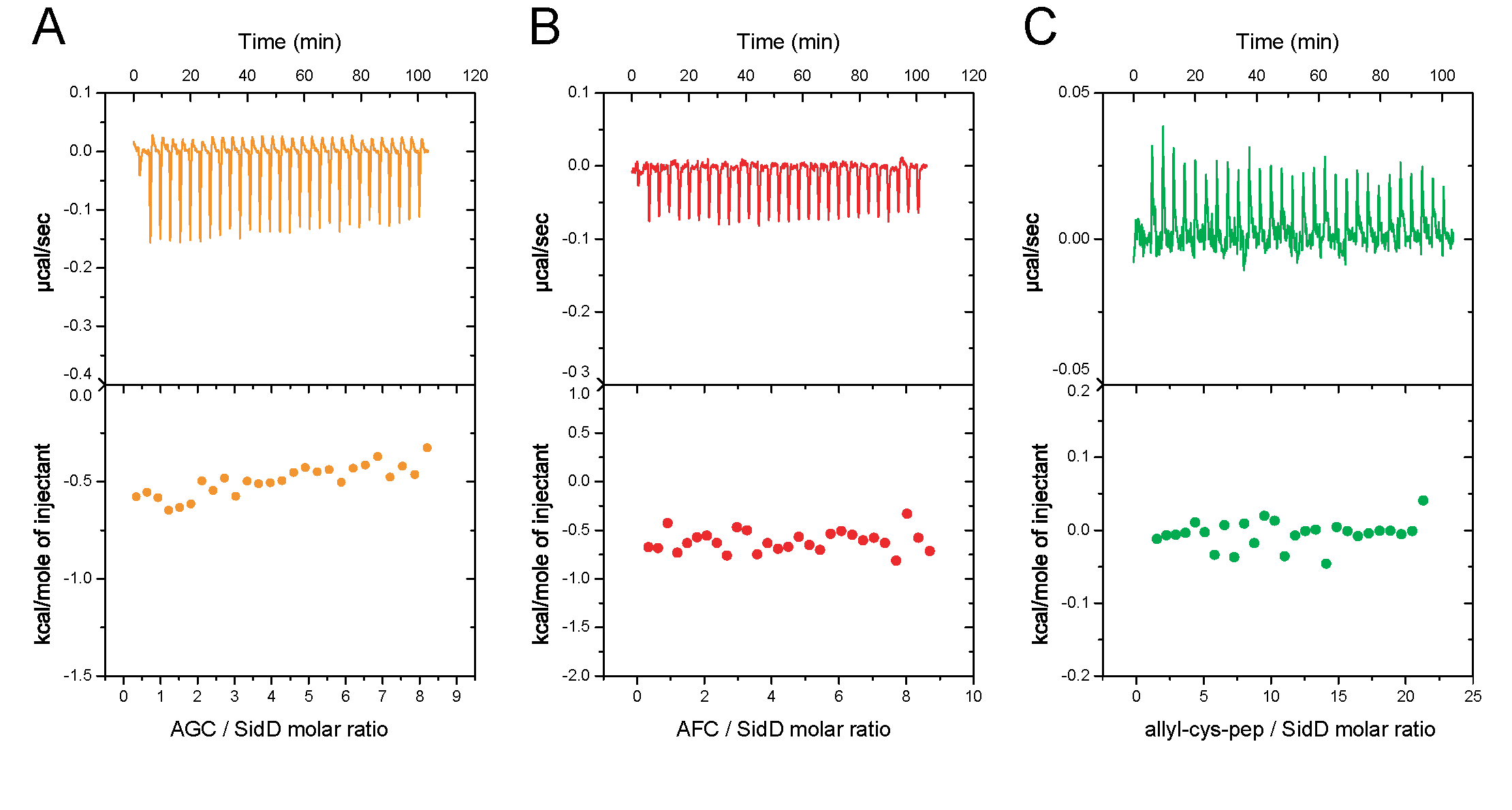

Supplement: S10 Fig — (TIF) [file ppat.1008734.s010.tif]

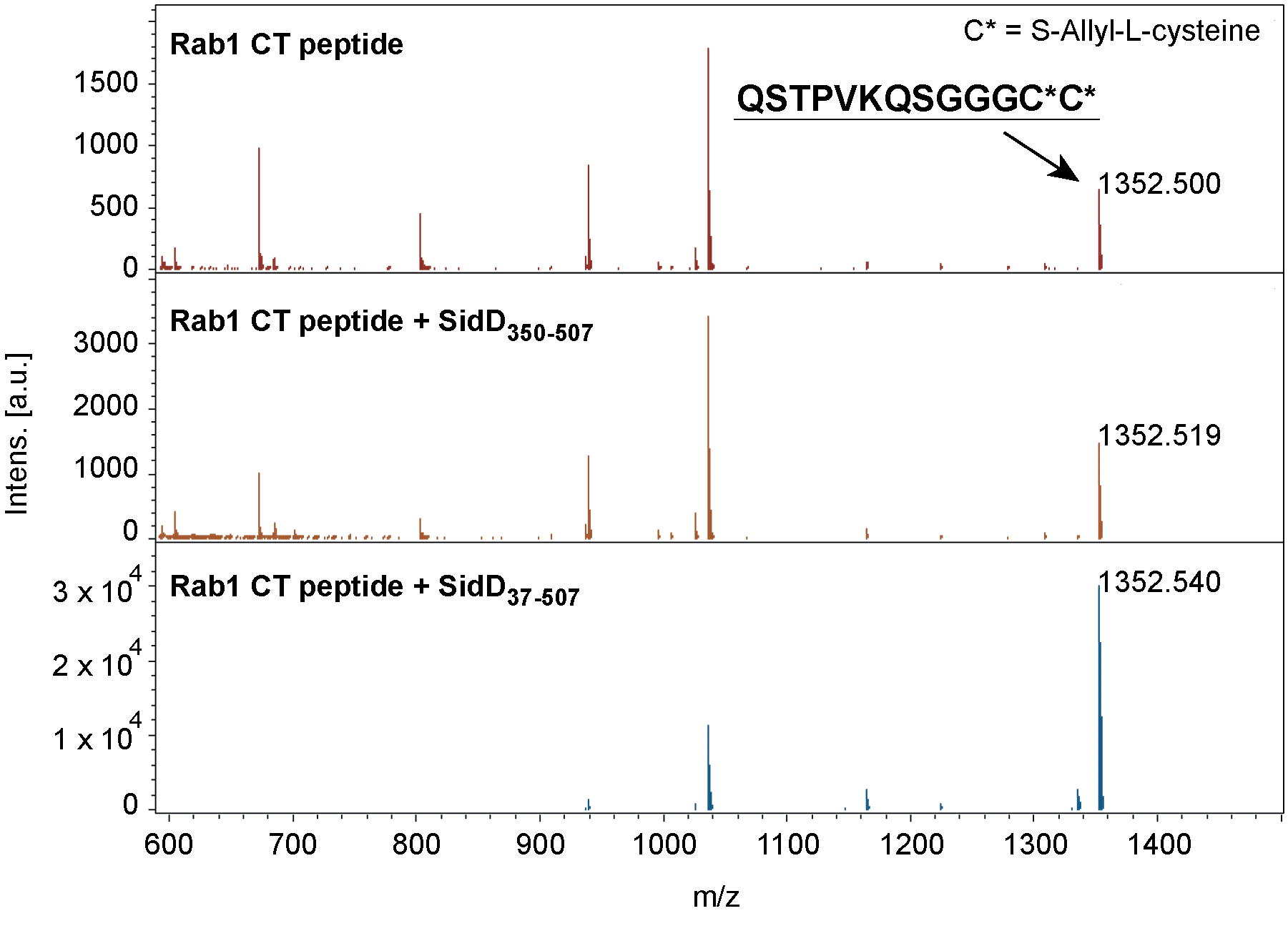

Supplement: S11 Fig — (TIF) [file ppat.1008734.s011.tif]

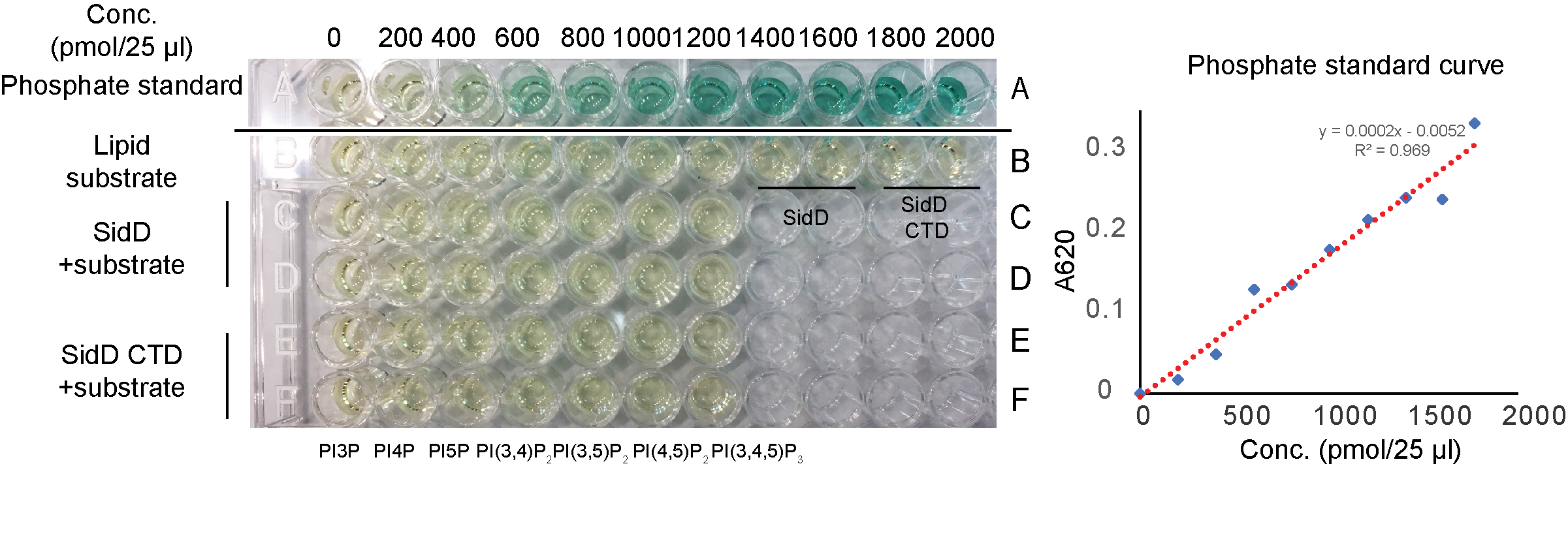

Supplement: S12 Fig — (TIF) [file ppat.1008734.s012.tif]

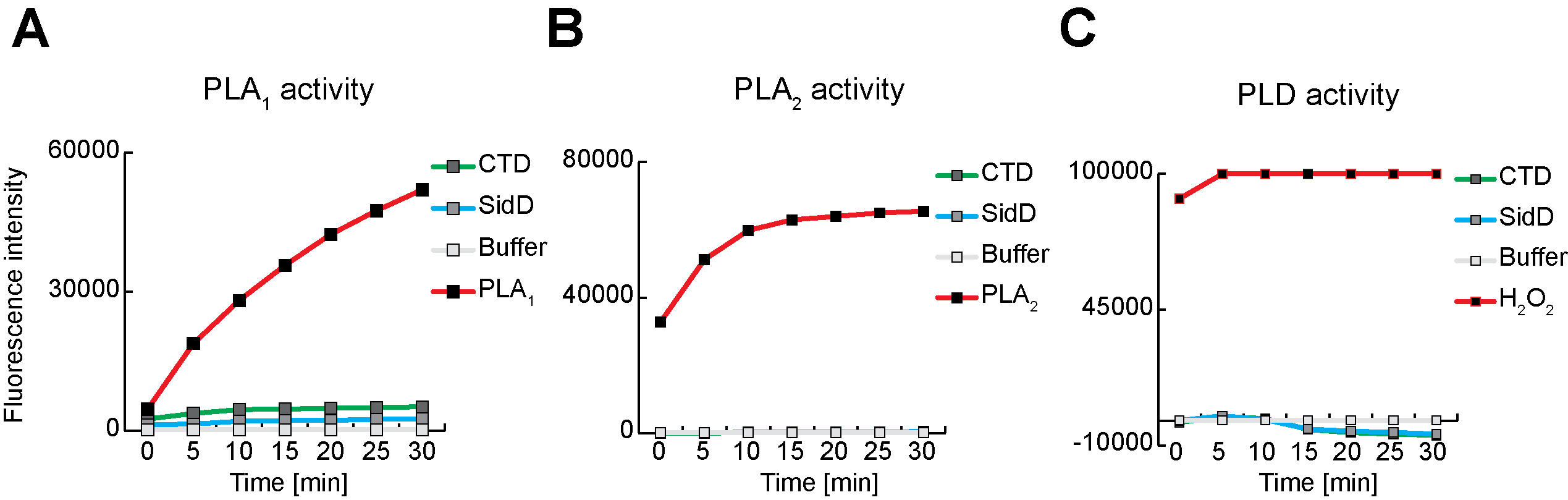

Supplement: S13 Fig — (TIF) [file ppat.1008734.s013.tif]
