# Supplementary material for: Structural insight into the membrane targeting domain of the Legionella deAMPylase SidD
Source: PLoS Pathog. 2020 Aug 27;16(8):e1008734. doi: 10.1371/journal.ppat.1008734 (PMC7480848; doi:10.1371/journal.ppat.1008734)
Supplement: S1 Table — (DOCX) [file ppat.1008734.s014.docx]

|  | **SidD _370-507_** | **SidD _370-507_** | **SidD _370-507_** | **SidD _37-507_** |
| --- | --- | --- | --- | --- |
|  | **Native** | **SeMet** | **Native-Zn** | **Native** |
| **Data collection** |  | |  |  |
| Wavelength [Å] | 0.9334 | 0.9796 | 1.2821 | 0.99996 |
| Space group | P63 | P63 | P63 | P21 |
| Resolution [Å] | 50-2.5 (2.6-2.5) | 50-2.7 (2.9-2.7) | 50-2.81 (2.98-2.81) | 50-3.8 (3.8-3.6) |
| Cell dimensions  *a, b, c* [Å]  α, β, γ [º] | 165.57, 165.57, 68.71  90, 90, 120 | 164.55, 164.55, 69.00  90, 90, 120 | 164.68, 164.68, 68,72  90, 90, 120 | 63.57, 141.64, 234.30  90.00, 92.75, 90.00 |
| CC_1/2_ (%) | 99.9 (54.4) | 100.0 (33.0) | 100.0 (48.9) | 99.8(61.0) |
| Completeness (%) | 99.8 (99.3) | 99.5 (98.2) | 99.6 (99.3) | 98.8 (95.6) |
| I/σ | 20.21 (1.37) | 18.81 (0.69) | 18.51 (1.06) | 6.0 (0.91) |
| Number of unique reflexions | 37978 (6069) | 57164 (9131) | 50772 (8232) | 48162 (7457) |
| Redundancy | 10.1 (10.1) | 9.8 (6.2) | 10.0 (7.9) | 3.38(3.4) |
| Alpha twin | 0.478 |  |  |  |
| **Refinement** |  | |  |  |
| R-factor (%) | 26.4 |  |  | 29.5 |
| R-free (%) | 28.7 |  |  | 33.8 |
| No. atoms |  |  |  |  |
| Waters | 58 |  |  | 1 |
| Ions | 11 |  |  | 4 |
| Ligand/glycerol | 4 |  |  | 0 |
| R.m.s deviations |  |  |  |  |
| Bond lengths (Å) | 0.004 |  |  | 0.003 |
| Bond angles (º) | 0.715 |  |  | 0.74 |
| **PDB CODE** | **6RP4** |  |  | **6RRE** |

**S1 Table. Data collection and refinement statistics**

*Highest resolution shell is shown in parenthesis.

** SeMet correspond to Selenomethionine labeled protein.
