# Supplementary material for: Structural insight into the membrane targeting domain of the Legionella deAMPylase SidD
Source: PLoS Pathog. 2020 Aug 27;16(8):e1008734. doi: 10.1371/journal.ppat.1008734 (PMC7480848; doi:10.1371/journal.ppat.1008734)
Supplement: S2 Table — (DOCX) [file ppat.1008734.s015.docx]

**S2 Table. Plasmids used in this study.**

| **Name** | **Insert** | **Oligonucleotides** | **Source or reference** |
| --- | --- | --- | --- |
| pGEX6p1-SidD CTD | *L. pneumophila* SidD 322-507 | BamH- SidD 322-endF SaII SidD 322-endR | This study |
| pEGFP-SidD CTD | *L. pneumophila* SidD 322-507 |  | [2] |
| pEGFP-SidD ∆loop | *L. pneumophila* SidD 322-507 ∆loop (deletion of aa 370-378) | 5sidD_del370-379 3sidD_del370-379 | This study |
| pEGFP-SidD CTD F370A | *L. pneumophila* SidD 322-507 F370A | 5SidDF370A 3SidDF370A | This study |
| pEGFP-SidD CTD F370S | *L. pneumophila* SidD 322-507 F370S | 5SidDF370S 3SidDF370S | This study |
| pEGFP-SidD CTD F370Y | *L. pneumophila* SidD 322-507 F370Y | 5SidDF370Y 3SidDF370Y | This study |
| pEGFP-SidD CTD Y374A | *L. pneumophila* SidD 322-507 Y374A | 5SidDY374A 3SidDY374A | This study |
| pEGFP-SidD CTD Y374S | *L. pneumophila* SidD 322-507 Y374S | 5SidDY374S 3SidDY374S | This study |
| pEGFP-SidD CTD Y374F | *L. pneumophila* SidD 322-507 Y374F | 5SidDY374F 3SidDY374F | This study |
| pEGFP-SidD CTD F376A | *L. pneumophila* SidD 322-507 F376A | 5SidDF376A 3SidDF376A | This study |
| pEGFP-SidD CTD F376S | *L. pneumophila* SidD 322-507 F376S | 5SidDF376S 3SidDF376S | This study |
| pEGFP-SidD CTD F376Y | *L. pneumophila* SidD 322-507 F376Y | 5SidDF376Y 3SidDF376Y | This study |
| pEGFP-SidD CTD F377A | *L. pneumophila* SidD 322-507 F377A | 5SidDF377A 3SidDF377A | This study |
| pEGFP-SidD CTD F377S | *L. pneumophila* SidD 322-507 F377S | 5SidDF377S 3SidDF377S | This study |
| pEGFP-SidD CTD F377Y | *L. pneumophila* SidD 322-507 F377Y | 5SidDF377Y 3SidDF377Y | This study |
| pEGFP-SidD CTD KK/EE | L. pneumophila SidD 322-507 K416E K433E | 5sidD_K416E  3sidD_K416E 5sidD_K433E 3sidD_K433E | This study |
| pEGFP-SidD CTD DE/RR | L. pneumophila SidD 322-507 D464R E467R | 5sidD_ED/RR 3sidD_ED/RR | This study |
| pEGFP-SidD 322-387 | *L. pneumophila* SidD 322-387 (Ala387stop) | 5' SidD 322-387 3' SidD 322-387 | This study |
| pEGFP-SidD 322-406 | *L. pneumophila* SidD 322-406 (Ile406stop) | 5'SidD 322-406 3'SidD 322-406 | This study |
| pEGFP-SidD 322-435 | *L. pneumophila* SidD 322-435 (Leu435stop) | 5'SidD 322-435 3'SidD 322-435 | This study |
| pEGFP-SidD 322-450 | *L. pneumophila* SidD 322-450 (Asn450stop) | 5' SidD 322-450 3' SidD 322-450 | This study |
| pEGFP-SidD 322-450 F370S | *L. pneumophila* SidD 322-450 F370S (Asn450stop F370S) | 5' SidD 322-450 3' SidD 322-450  5SidDF370S 3SidDF370S | This study |
| pNPTS138D-*sidD* flank | *L. pneumophila* SidD including flanking region | attB1_sidD_upst attB2_sidD_down | Lab resource |
| pNPTS138D-*sidD* ∆loop | *L. pneumophila* SidD including flanking region with deletion of sequence encoding SidD 370-378 | 5sidD_del370-379 3sidD_del370-379 | This study |
| pHis-Parralle2-SidD_37-507_ | *L. pneumophila* SidD 37-507 | SidD-37-NdeI-up  SidD-507-5His-BamHI-low | This study |
| pHis-Parralle2-SidD_350-507_ | *L. pneumophila* SidD 350-507 | SidD-350UP-BamHI  SidD-507low-ncoI-6his | This study |
| pHis-Parallel2-SidD_∆loop_ | *L. pneumophila* SidD 37-507 (∆ aa 370-378) | SidD∆loop-up  SidD∆loop-low  Ampi-up  Ampi-low | This study |
| pEGFP-CTD L469A | L. pneumophila SidD 322-507 L469A | 5’sidDL469A  3’sidDL469A | This study |
| pEGFP-CTD L469A/K472A | *L. pneumophila* SidD 322-507 L469A/K472A | 5’sidDL469A/K472A  3’sidDL469A/K472A | This study |
| pEGFP-CTD D484A | *L. pneumophila* SidD 322-507 D484A | 5’sidDD484A  3’sidDD484A | This study |
| pEGFP-CTD D484A/I495A | *L. pneumophila* SidD 322-507 D484A/I495A | 5’sidDD484A/I485A  3’sidDD484A/I485A | This study |
| pEGFP-CTD L469A/K472A/ D484A/I495A | *L. pneumophila* SidD 322-507 L469A/K472A/ D484A/I495A | 5’sidDD484A/I485A  3’sidDD484A/I485A | This study |
