# Supplementary material for: Structural insight into the membrane targeting domain of the Legionella deAMPylase SidD
Source: PLoS Pathog. 2020 Aug 27;16(8):e1008734. doi: 10.1371/journal.ppat.1008734 (PMC7480848; doi:10.1371/journal.ppat.1008734)
Supplement: S3 Table — (DOCX) [file ppat.1008734.s016.docx]

**S3 Table. Oligonucleotides used in this study.**

| **Primer name** | **Primer sequence** |
| --- | --- |
| attB1_sidD_upst | GGGGACAAGTTTGTACAAAAAAGCAGGCTTCCGGCATCTCGACACCAAGCGCGTCAAAGG |
| attB2_sidD_down | GGGGACCACTTTGTACAAGAAAGCTGGGTCGGCTCACCTTTTGGTGATGATCAAGTAGG |
| 5sidD_del370-379 | CTTTCATACATCAAGAATGAAAATGAAAACAAAGGT GGAGCTGATAAAAATATAGAAA |
| 3sidD_del370-379 | TTTCTATATTTTTATCAGCTCC ACCTTTGTTTTCATTTTCATTCTTGATGTATGAAAG |
| 5SidDF370A | GAAAATGAAAACAAAGGTGCTCTTGGTATATATGG |
| 3SidDF370A | CCATATATACCAAGAGCACCTTTGTTTTCATTTTC |
| 5SidDF370S | GAAAATGAAAACAAAGGTAGTCTTGGTATATATGG |
| 3SidDF370S | CCATATATACCAAGACTACCTTTGTTTTCATTTTC |
| 5SidDF370Y | GAAAATGAAAACAAAGGTTATCTTGGTATATATGG |
| 3SidDF370Y | CCATATATACCAAGATAACCTTTGTTTTCATTTTC |
| 5SidDF376A | CTTGGTATATATGGTGCCTTCACAGGAGCTGA |
| 3SidDF376A | TCAGCTCCTGTGAAGGCACCATATATACCAAG |
| 5SidDF376S | CTTGGTATATATGGTAGCTTCACAGGAGCTGA |
| 3SidDF376S | TCAGCTCCTGTGAAGCTACCATATATACCAAG |
| 5SidDF376Y | CTTGGTATATATGGTTACTTCACAGGAGCTGA |
| 3SidDF376Y | TCAGCTCCTGTGAAGTAACCATATATACCAAG |
| 5SidDF377A | GGTATATATGGTTTCGCCACAGGAGCTGATAA |
| 3SidDF377A | TTATCAGCTCCTGTGGCGAAACCATATATACC |
| 5SidDF377S | GGTATATATGGTTTCAGCACAGGAGCTGATAA |
| 3SidDF377S | TTATCAGCTCCTGTGCTGAAACCATATATACC |
| 5SidDF377Y | GGTATATATGGTTTCTACACAGGAGCTGATAA |
| 3SidDF377Y | TTATCAGCTCCTGTGTAGAAACCATATATACC |
| 5SidDY374F | GGTTTTCTTGGTATATTTGGTTTCTTCACAGG |
| 3SidDY374F | CCTGTGAAGAAACCAAATATACCAAGAAAACC |
| 5SidDY374S | GGTTTTCTTGGTATATCTGGTTTCTTCACAGG |
| 3SidDY374S | CCTGTGAAGAAACCAGATATACCAAGAAAACC |
| 5SidDY374A | GGTTTTCTTGGTATAGCTGGTTTCTTCACAGG |
| 3SidDY374A | CCTGTGAAGAAACCAGCTATACCAAGAAAACC |
| 5sidD_K416E | CTGCTCTAGTCTCTGATAGTGAGACACCCCTAATGACTCAG |
| 3sidD_K416E | CTGAGTCATTAGGGGTGTCTCACTATCAGAGACTAGAGCAG |
| 5sidD_K433E | GGCTATCTGGATTTCCCGTCGGAGGCATTACTTGCTAATAAAATTAC |
| 3sidD_K433E | GTAATTTTATTAGCAAGTAATGCCTCCGACGGGAAATCCAGATAGCC |
| 5sidD_ED/RR | CTGGGTTCCAGATTAGCAACTCGTGTTATACGGGAATTAGAAACTAAAATTATTC |
| 3sidD_ED/RR | GAATAATTTTAGTTTCTAATTCCCGTATAACACGAGTTGCTAATCTGGAACCCAG |
| 5' SidD 322-387 | GGAGCTGATAAAAATATAGAAAAAGCGTAAGTCGACGGTACCGCGGGCCCGGGATCC |
| 3' SidD 322-387 | GGATCCCGGGCCCGCGGTACCGTCGACTTACGCTTTTTCTATATTTTTATCAGCTCC |
| 5'SidD 322-406 | AATAATCATTTTATCTCTCTAATTTAAGTCGACGGTACCGCGGGCCCGGGATCC |
| 3'SidD 322-406 | GGATCCCGGGCCCGCGGTACCGTCGACTTAAATTAGAGAGATAAAATGATTATT |
| 5'SidD 322-435 | CTGGATTTCCCGTCGAAAGCATTATAAGTCGACGGTACCGCGGGCCCGGGATCC |
| 3'SidD 322-435 | GGATCCCGGGCCCGCGGTACCGTCGACTTATAATGCTTTCGACGGGAAATCCAG |
| 5' SidD 322-450 | GAATTGCTTTTAAAAGAATTAGAAAATTAAGTCGACGGTACCGCGGGCCCGGGATCC |
| 3' SidD 322-450 | GGATCCCGGGCCCGCGGTACCGTCGACTTAATTTTCTAATTCTTTTAAAAGCAATTC |
| BamH SidD 322-endF | CG GGATCC CAACGTTTTGGT |
| SaII SidD 322-endR | ACGC GTCGACTTAAATAGTAAG |
| SidD-37-NdeI-up | TCCGGACCATGGGAATGCTGTCGATTATTACACAAATCTG |
| SidD-507-5His-BamHI-low | TCCGGAGGATCCTTAGTGGTGGTGGTGGTGAATAGTAAGACTCGAGTTAGTTGCAGCC |
| SidD-350UP-BamHI | TCCGGAGGATCCTCAATCGGAGTAAGTGATGGATTACTTTCATAC |
| SidD-507low-ncoI-6his | GATAAAATTAAGGCTGCAACTAACTCGAGTCTTACTATTCACCATCACCATCACTGACCATGGTCCGGA |
| SidD∆loop-up | GAAAATGAAAACAAAGGTGGAGCTGATAAAAATATAGAAAAAGCGACTC |
| SidD∆loop-low | CTATATTTTTATCAGCTCCACCTTTGTTTTCATTTTCATTCTTGATGTATGAAAGTAATCC |
| Ampi-up | GACAGTAAGAGAATTATGCAGTGCTGCCATAACCATGAG |
| Ampi-low | CTCATGGTTATGGCAGCACTGCATAATTCTCTTACTGTC |
| 5’sidDD484A | aacccagcaggctcg gCt attcattcaacgctaaac |
| 3’sidDD484A | gtttagcgttgaatgaataGccgagcctgctgggtt |
| 5’sidDD484A/I485A | aacccagcaggctcg gCt GCtcattcaacgctaaac |
| 3’sidDD484A/I485A | gtttagcgttgaatgaGCaGccgagcctgctgggtt |
| 5’sidDL469A | gatgttatagaggaa GCa gaaactaaaattatt |
| 3’sidDL469A | aataattttagtttctGCttcctctataacatc |
| 5’sidDL469A/K472A | gaggaaGCa gaaact GCa attattcgttatatt |
| 3’sidDL469A/K472A | aatataacgaataattGCagtttctGCttcctc |
